# COVID-19 Contact Tracing: Challenges and Future Directions

**DOI:** 10.1109/ACCESS.2020.3036718

**Published:** 2020-11-09

**Authors:** Mohammad Jabed Morshed Chowdhury, Md Sadek Ferdous, Kamanashis Biswas, Niaz Chowdhury, Vallipuram Muthukkumarasamy

**Affiliations:** Department of Computer Science and Information TechnologyLa Trobe University 2080 Melbourne VIC 3086 Australia; Department of Computer Science and EngineeringShahjalal University of Science and Technology 113074 Sylhet 3114 Bangladesh; Centre for Global Finance and TechnologyImperial College London 4615 London SW7 2AZ U.K.; Peter Faber Business SchoolAustralian Catholic University 95359 North Sydney NSW 2060 Australia; Knowledge Media Institute, The Open University 5488 Milton Keynes MK7 6AA U.K.; School of Information and Communication TechnologyGriffith University 5723 Gold Coast QLD 4215 Australia

**Keywords:** COVID-19, contact tracing, privacy, proximity technologies

## Abstract

Contact tracing has become a vital tool for public health officials to effectively combat the spread of new diseases, such as the novel coronavirus disease COVID-19. Contact tracing is not new to epidemiologist rather, it used manual or semi-manual approaches that are incredibly time-consuming, costly and inefficient. It mostly relies on human memory while scalability is a significant challenge in tackling pandemics. The unprecedented health and socio-economic impacts led researchers and practitioners around the world to search for technology-based approaches for providing scalable and timely answers. Smartphones and associated digital technologies have the potential to provide a better approach due to their high level of penetration, coupled with mobility. While data-driven solutions are extremely powerful, the fear among citizens is that information like location or proximity associated with other personal data can be weaponised by the states to enforce surveillance. Low adoption rate of such apps due to the lack of trust questioned the efficacy and demanded researchers to find innovative solution for building digital-trust, and appropriately balancing privacy and accuracy of data. In this paper, we have critically reviewed such protocols and apps to identify the strength and weakness of each approach. Finally, we have penned down our recommendations to make the future contact tracing mechanisms more universally inter-operable and privacy-preserving.

## I. Introduction

The novel corona virus disease 2019 (COVID-19) pandemic has created a public health crisis, with epidemiological models predicting severe consequences, including unprecedented death rates. Due to its high infectious rate (denoted with $R_{0}$ and pronounced as “R-naught”), health professionals around the world have advised to maintain hygiene and social distancing [1]. $R_{0}$ is the expected number of cases directly generated by one infected case in a population where all individuals are susceptible to infection [2]. Health departments around the world use a manual process to track all the people who came in contact of a COVID-19 affected person within last 14 to 21 days. This process is extremely time consuming, inefficient, highly error prone and not scalable. Therefore, governments are turning to the digital technologies and data analytics for a better solution [3]. Contact tracing using smartphone technology seems to be a powerful tool that may be employed to collect data and to limit disease transmission during an epidemic or pandemic [4]. Singapore, Australia, China and India along with a few other countries are early adopters of mobile based contacting tracing apps as a mechanism for softening the lock down, which creates enormous economic crisis.

Though these apps automate the proximity tracing quite efficiently they present significant privacy concerns regarding the collection of sensitive data such as personal interactions and locations. Privacy protection laws around the world, such as GDPR (Global Data Protection Regulation) [5] and local data protection regulations are still in effect during this emergency. This implies that those who create and/or roll out these tools still have to demonstrate good governance of the data being collected, transmitted, stored, shared and analysed.

As more states and organisations are searching for contact tracing or exposure notification tools for a COVID-safe society/workplace, it is vital to clearly appreciate different aspects of such mechanisms and understand the dynamics, particularly security and privacy. These may have serious implications on individual’s safety, social stigma and societies civil rights. Towards this aim, there have been a few attempts to explore and analyse different contact tracing applications. In [6], the authors have outlined different technological approaches to mobile-phone based contact-tracing and highlighted the risks these apps pose to individuals and societies. They have recommended the security enhancing approaches that can mitigate these risks and described trade-offs one must make when developing and deploying any mass contact-tracing technology.

The authors in [7] have presented a detailed analysis of different privacy aspects in contact tracing applications. The analysis is specifically based on the *TraceTogether app*, a contact tracing mobile app facilitated by the Government of Singapore [8]. In this article, the authors have identified privacy lapses in TraceTogether and explored different approaches to improve these privacy lapses which are reviewed below. Li and Guo have surveyed different contact tracing applications and protocols deployed around the world [9]. They have presented a brief discussion about different technologies or techniques used in contact tracing app, such as QR (Quick Response) code, big data, Bluetooth, GPS (Global Positioning System) and WiFi (Wireless Fidelity). They have identified the challenges and research directions for Bluetooth based contact tracing. Ahmed *et al.* have conducted a survey on the COVID-19 contact tracing apps based on few key attributes such as system architecture, data management, privacy, security, proximity estimation, and attack vulnerability [10]. They have also presented an overview of many contract tracing apps. Finally, they have advocated for the improvement in proximity accuracy, use of decentralised architecture and artificial intelligence-based algorithms in aiding the decision making process.

Overall, different authors have touched on different aspects of the contact tracing application. However, to the best of our knowledge, none of the existing works has been able to cover the whole spectrum of different properties corresponding to a contact-tracing app, such as the proximity technologies, protocols, application, security, privacy and universal coverage. In this article we aim to fill in this gap. In particular, we have made the following contributions in this article:

1) Critically reviewed the proximity measuring technologies.
2) Analysed the limitations of different contact tracing protocols.
3) Developed a taxonomy covering the full spectrum of factors that a contact tracing app should consider.
4) Reviewed and compared various contact tracing applications.
5) Proposed possible models for unified operations in future.

The article is structured as follows. Section II has discussed about different proximity measurement technologies. It is followed by a thorough analysis of contact tracing protocols (along with a taxonomy of evaluation matrices) and review of contract tracing apps, presented in section III and IV respectively. In section V, we have presented an analysis of our review and a series of recommendations. Finally, we have concluded in section VI.

## II. Proximity Measurement Technologies

Detecting accurate human proximity is important for planning and operation in many disciplines, such as social science, architecture and health. Measuring proximity mainly relies on the phenomenon being observed. In certain cases, only a brief interaction between two people considered as close proximity, whereas, in some cases, it requires prolonged interactions to be treated as meaningful proximity encounter.

In terms of COVID-19, 1.5 meters of proximity with sufficient exposure to any COVID-19 patient may result in infection [11]. Therefore, correctly estimating the distance between two people and the duration of exposure is vital. Different technologies such as GPS [12], Bluetooth [13] and WiFi [14] help to estimate the physical distance and the extent of interactions. In this section, we will briefly discuss these technologies and compare them against a few properties, such as granularity, location privacy and range. This will assist any contact tracing protocol/app designer to choose the appropriate technologies.

### A. GPS-Based Proximity Tracking

The GPS is probably the most popular technology that we are familiar when comes to location information. It is a network of about 30 satellites orbiting the Earth at an altitude of 20, 000 km. Individuals carrying the GPS receivers capture GPS signal which is transmitted by the GPS satellites. The receiver calculates the time delay of each of the received signal, which is a measure of the distance to each of the satellites. Once it has this information from four satellites the receiver can pinpoint our location using a process called *trilateration*. GPS locations are often used to calculate proximity between two objects [15], [16]. Khoroshevsky and Lerner have also used GPS to discover human mobility-pattern discovery and next-place [17]. Mobile applications widely use GPS to provide localised services [18]. Therefore, it is not surprising to see a number of the COVID-19 tracing application use GPS to calculate proximity.

GPS calculates and identifies someone’s location using longitude and latitude coordinate. Though GPS is one of the most popular means to find someone’s location or calculate proximity, it is not the most efficient one. It cannot calculate precise locations in indoors. Typical accuracy in GPS calculation is about 5 meters [19]. Due to the signal attenuation caused by construction materials, the satellite based GPS loses significant power indoors affecting the required coverage. In addition, the multiple reflections at surfaces cause multi-path propagation issues causing uncontrollable errors. Moreover, it captures the absolute location of individual which is a threat for location privacy and can lead to surveillance.

### B. Bluetooth-Based Proximity Tracking

Bluetooth is an important candidate for wireless localisation on consumer smart devices such as smartphone. The traditional Bluetooth has significantly long scan time (10 s), which limits its value for localisation. However, the new protocol called, Bluetooth Low Energy (BLE), supported by most smart devices since 2015, has overcome this limitation. Bluetooth has many advantages such as small size, light weight, low cost, power saving and widely supported by smart devices. Therefore, BLE has become a dominant wireless proximity technology. In the BLE protocol definition, 40 channels, each 2 MHz wide at the 2.4 GHz ISM band, are used to transmit messages [20]. The duration for transmitting messages is extremely short to save battery power. Among these 40 channels, there are three channels (i.e., 37, 38, and 39) for broadcasting advertisement messages. The Received Signal Strength Indicator (RSSI) from these three channels can be used for estimating the target’s proximity. The BLE advertising rate can be set up to 50 Hz. The transmission power for BLE beacons are also set from 0 dBm to −75 dBm. To reduce power consumption, BLE advertising rate and transmission power are usually set to less than 10 Hz and −16 dBm, respectively [21]. Here, dBm indicates decibel-milliwatts (dBm) with which the RSSI is measured, the higher the RSSI number, the stronger the signal is. The equation (1) shows the relationship between RSSI measurement and distance. In this formula n is the propagation constant or path-loss exponent and d is the distance in meters. A is the received signal strength in dBm at 1 meter distance [22].

\begin{equation*} \text {RSSI(dBm)} = -10n {~\text {log}}_{10} (d) + A\tag{1}\end{equation*}

Mobile apps which use bluetooth use the RSSI values to calculate the distance between two individuals. The reading for RSSI values also vary between different mobile phone models and operating systems. The app needs to factor that variations to get the appropriate distance. In addition, the app also needs to calculate the amount of time, two individuals are in close contact. Bluetooth devices do not capture the absolute location of any individual rather it records if there is any Bluetooth devices within the radio range. It alone cannot reveal where that interaction has happened. Therefore, it provides more safeguard against location privacy.

### C. WiFi Based Positioning

A standard WiFi based positioning system, such as the one offered by Cisco, generally utilises access points installed in a facility and radio transceivers already present in the user devices. These standard WiFi based positioning systems can realise any location-aware application that involves PDAs (Personal Digital Assistant), laptops, bar code scanners, voice-over-IP phones and other 802.11 enabled devices [23]. Without the need for additional hardware, institutions or businesses can install the system much faster and significantly reduce the overhead costs. A common infrastructure supports both data network and positioning system where the latter works wherever there is WiFi coverage. WiFi location positioning operates on a grid of WiFi hotspots providing, in general, with 20 – 30 meters of location accuracy [24].

Nowadays, more sophisticated software-based hybrid approaches can also offer better accuracy [25], [26]. For example, the use of Kalman Filters can reduce time delays upon location fingerprinting for point data, collection in the presence of WiFi network where GPS fails to provide adequate service [27], [28]. Particle filters, on the other hand, can be used for integrated navigation in aircraft and cars [29]. Several smart systems are also available in the literature that works in conjunction with WiFi location tracking and users’ movement to identify their location [30]; for example, the intelligent fusion algorithm that makes the use of moving direction information without requiring any user intervention [31], [32].

### D. Cellular Network Based Location Calculation

We can view a typical cellular network as being composed of a number of base transceiver stations (BTS) belonging to a location area code (LAC) and connected to a core network [33]. The central network contains a home location register (HLR) that keeps track of each mobile station’s last known location. As a mobile phone moves with its user, the phone pings nearby cell towers or cell sites. This process generates location information, about the cell towers to which the phone has sent a signal, which is stored by the telecommunication operators (“Telcos”). With proximity information from multiple cell towers, a technique called *triangulation* is used to estimate the location of a cell phone with greater precision [34]. Governments can compel Telcos to provide that mobile location information to track someone’s real time or past movement.

### E. Other Technologies and Techniques

Other than the above mentioned technologies, there are a few other technologies such as RFID (Radio-Frequency Identification) II-E1 and NFC (Near Field Communication) II-E2 which may be used in contact tracing in different dimensions. For instance, QR code based contact tracing is used in Chinese apps [35]. RFID and NFC can be used to replace QR code based contact tracing scenarios, such as in workplaces or sporting events.

#### 1) Radio-Frequency Identification (RFID)

RFID uses electromagnetic fields to automatically identify and track tags attached to objects. It is widely used in libraries, supply chain and retail stores. There are passive, semi-passive, and active RFIDSs equipped with transponders [36]. The passive system uses simple, battery-free tags and high-power readers [37]. These are often used for tracking assets through a check point and for anti-theft efforts. The tags are powered by the RF emitted by the base, the *range is usually in inches*. Therefore, it is quite unusable for COVID-19 proximity tracking, because the proximity range is 1.5 to 2 meters.

#### 2) Near Field Communication (NFC)

NFC is an extremely short range technology, typically 4 to 10cm. NFC is a wireless protocol designed to be a replacement for plugging a cord between two devices [38]. Instead of taking out a cable, you get the two devices within the very short range of NFC communication, and let them find and communicate with each other wirelessly [39]. This technology is widely used for contactless payments. The typical range of the NFC chip makes it unusable for contact tracing applications.

### F. Discussion

In this section, we will compare different proximity measurement technologies to assist contact tracing protocol/app developers or policy makers to decide on the technology. We have evaluated the technologies based on a few properties relevant for COVID-19 and privacy regulations. *Location accuracy* discusses how accurately it can detect the proximity between two particular devices. *Energy consumption* provides information about the battery performance of the used technology. *Privacy* highlights whether it captures the absolute location or only proximity information. Many of these technologies cannot differentiate that two people are living in two different floors in a building or separated by a brick wall and thus, can provide false positive of proximity.

## III. Contact Tracing Protocols

In this section, we review the underlying methodologies of a number of contact tracing protocols. In total, we have reviewed 12 protocols. In Section III-N, we also compare these protocols against the evaluations matrices of our taxonomies.

### A. TCN

Temporary Contact Numbers (TCN) Protocol [4] is a decentralised privacy-preserving contact tracing protocol initiated by TCN Coalition, a global community of technologists. The protocol specification, written and maintained in a non-formal way on Github, aims to achieve three main goals: privacy, capacity and integrity. The semantics of these goals along with a number of sub-goals and other aspects are discussed below.

• Actors: The protocol assumes three different categories of users: Server (an authority); Reporters who submit reports (discussed later) to the server; and Receivers who receive reports from the server.
• Privacy: the specification considers different levels of privacy as outlined below:
  • Reporter privacy implies that a reporter does not reveal information to any user other than the contacts and only reveals the time of contact to the contacts.
  • Receiver privacy implies that a receiver should not reveal any information to others.
  • Server privacy implies that an honest-but-curious server cannot infer any information regarding the location or the contacts of a reporter.
  • No passive tracking indicates the scenario in which an adversary monitoring Bluetooth connections cannot deduce the location of a receiver.
• Capacity: It indicates that the system should have low technical barriers for adoption and the authority should be able to maintain the system with resilience.
• Integrity: The protocol considers different types of integrity:
  • Report integrity ensures the integrity of the reports.
  • Source integrity indicates that a reporter cannot send reports to non-contacting receivers (with whom the reporter did not come in contact).
  • Broadcast integrity implies that a reporter cannot submit TCNs that they did not generate.

**FIGURE 1.**
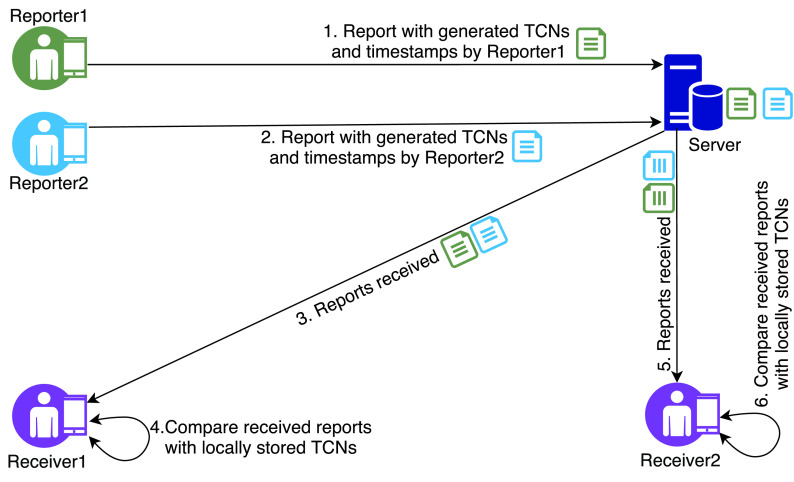
TCN protocol.

The TCN protocol is illustrated in Figure 1 and discussed next. Each mobile device equipped with the app running the TCN protocol generates and stores temporary numbers (known as *Temporary Contact Numbers* or *TCN*) at different intervals which are then broadcast using Bluetooth. When two users running the apps come in close contact with each other, they exchange their corresponding TCN numbers valid for that time-period and store them in their mobile devices. A reporter (presumably after being diagnosed with COVID-19) generates a report consisting of locally generated TCNs and their corresponding timestamps and submits the report to a server. Once a new report is uploaded, the server notifies the apps of all users. Then, the receivers can download the newly uploaded report to their respective mobile app and compare it with their list of received TCNs from other contacts. If there is a match found, it indicates that the receiver has a positive contact with the reporter.

The authors of the TCN protocol specification argue that broadcast integrity is hard to address as anyone can include the TCNs generated by other users in their report. The only way to mitigate this is by using a hardware level authentication. Similarly, the protocol cannot provide source integrity as anyone, not only the contacts, can download the reports. Other than these two issues, the authors argue that their protocol satisfies all other goals. However, the proposal also has a scalability issue as a receiver might need to download a significant amount of reports during a time period, depending on the severity of infections. The authors have proposed to generate TCNs for each report using a seed data which can reduce the number of TCNs within a report and thus solve the scalability issues to an extent.

### B. Epione

**FIGURE 2.**
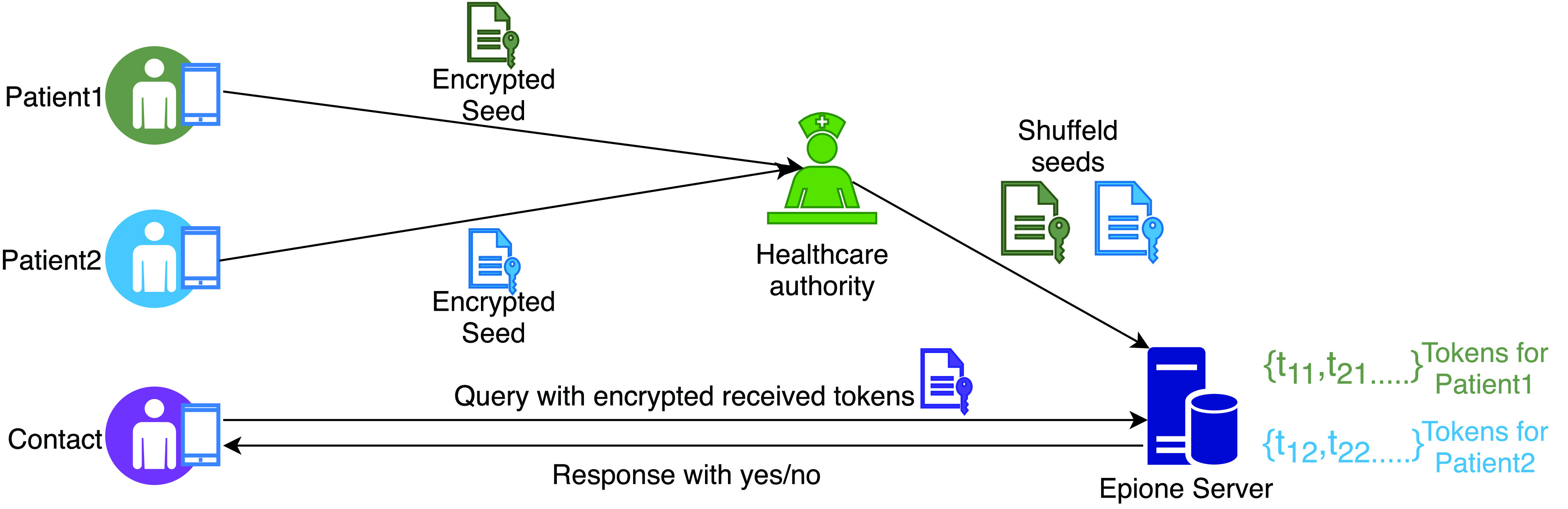
Epione protocol.

In [40], the authors have proposed *Epione* - a decentralised contact tracing app supporting a novel protocol (Figure 2) with a strong guarantee of privacy. To achieve this privacy guarantee, Epione utilises Private Set Interaction Cardinality (PSI-CA). Private Set Interaction (PSI) is a cryptographic mechanism which relies on multi-party computation (MPC). PSI enables two parties holding two different sets of data to compare the encrypted versions of these data sets in order to compute the size of their intersection, without revealing the contents of their set to the other party [41]. PSI-CA is a restrictive version of PSI in which even the intersection set is not revealed, only the cardinality of the intersection set is computed.

In Epione, there are three parties: users, a healthcare provider and Epione server. Users need to install a mobile app, also called Epoine, with which the users participate in the protocol. The healthcare provider can validate if a user is diagnosed with COVID-19 whereas the Epione server is the entity where the required computation for PSI-CA is carried out.

At the initial stage, the Epione server generates a public/private key pair which is supplied to each newly installed and registered app. Then, it generates a random seed which is used to generate random tokens at different time periods. The tokens are generated in such a way that a single token for a particular time period can be re-generated in the Epione server once the server knows the random seed. In the protocol, when a user comes to the close proximity of a contact, their apps exchange the respective tokens with each other using Bluetooth, which are stored in two separate lists in each app: “sent token list” and “received token list”.

When a user is diagnosed positive for COVID-19 (as confirmed by the healthcare provider), the user encrypts the respective random seed with the public key of Epione server, it is then submitted to the healthcare provider. The provider collects a number of such encrypted seeds from different users and shuffles them and transfers the set of seeds to the Epione server. In this way, the server has no knowledge about the original source of the encrypted seeds. The server then re-generates the tokens for each seed at its end, signifying the list of tokens disseminated by each infected user during a time period.

When a user would like to query if she has been in contact with any COVID-19 diagnosed patient, the user sends a query to the Epione server. The query is accompanied by a list of encrypted tokens received by the user during a particular time period. These tokens being encrypted means that the server has no knowledge of the contents of the tokens. However, the PSI-CA protocol is enacted to create a private set intersection between the set of transmitted encrypted tokens and the encrypted tokens generated at the server. The protocol then returns the cardinality (the number of elements) of the intersection set. A cardinality of greater than 0 implies that the user has been in contact with a COVID-19 patient and she should act accordingly.

The authors have formally proven the security of their protocol and analysed how their protocol mitigates a number of privacy issues such as linkage attacks by the server, linkage attacks by other users, user tracking and identification and malicious user queries. With all these features, Epione is one of the most robust privacy-preserving contact tracing protocols proposed. However, its main limitation is the difficult adoption barrier as the server needs to employ strong cryptographic mechanisms which cannot be easily deployed using off-the-shelf tools and hence, would require cryptographic experts to get involved during the adoption process. Unfortunately, such specialists might not be available in all situations/regions which could limit any wide-scale adoption. Another attack vector against Epione is as follows. Since PSI-CA would require the server to engage in heavy computations, a DoS (Denial of Service) attack against the server can be effectively launched by generating a large number malicious queries by colluding attackers. The authors did not consider this possibility and therefore, proper countermeasures must be considered to mitigate this attack.

### C. MPC Protocol

**FIGURE 3.**
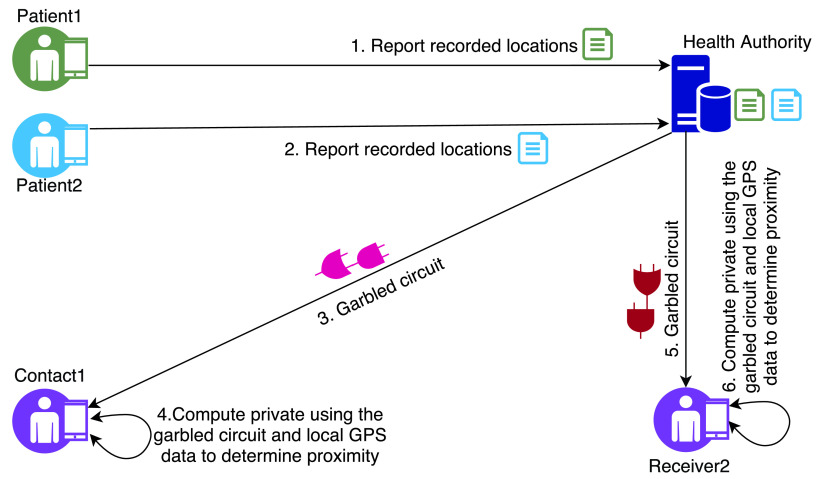
Reichert *et al.* protocol.

In [42], Reichert *et al.* have proposed a theoretical privacy-preserving approach for contact tracing which leverages Multi-party computation (MPC). In their proposal (Figure 3), there are two actors: users and Health Authority (HA). Users using their mobile devices record their GPS location data which are stored in their mobile devices along with the timestamps. Once a user (let us call the user *Alice*) is tested positive for COVID-19, the user shares this data set with the HA. When another user (called *Bob*) would like to check if he has ever been in contact with any COVID-19 patient, Bob interacts with the HA. The HA constructs a garbled circuit (a cryptographic protocol for two-party computation for evaluating a computational function over private data from two users [43]) using the data from Alice and sends it back to Bob. Bob then can use the circuit to privately evaluate, using Bob’s geo-location data, and compute if there is any geo-location point which will indicate a close contact with a positive patient.

The authors did not implement the protocol and therefore, its computational efficiency is unknown. In addition, the scalability of the proposal is also questionable as the HA will incur a significant computational complexity when the numbers of users will increase. This also opens up the possibility for launching DoS attacks against the HA when a number of users collude. Another issue is that, in this protocol, a COVID-19 patient shares her location data with the HA which can be used for linkage attacks against the patient.

### D. DP3T

Troncoso *et al.* [44] have proposed the Decentralised Privacy-Preserving Proximity Tracing (DP3T), a Bluetooth-based privacy-preserving contact tracing protocol that necessitates the safeguarding of personal and location data of the users. It ensures data minimisation by only allowing the central server to observe anonymous identifiers of infected people without any proximity information. This design principle of the protocol restricts authorities from learning the health condition of the individuals unless they willingly reach out, enabling epidemiologists to obtain minimal information regarding close contacts. In this protocol, no entity, including the backend server, can track non-infected users based on broadcast ephemeral identifiers.

**FIGURE 4.**
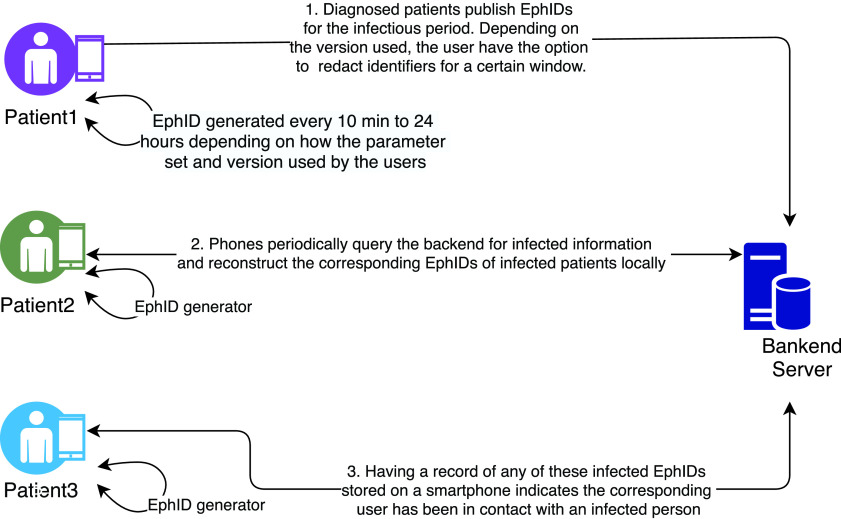
DP3T protocol.

The protocol has two versions designed to benefit two distinct scenarios. The first version is known as the low-cost decentralised proximity tracing, while the second is called unlinkable decentralised proximity tracing. The low-cost version has satisfying privacy properties and minimal bandwidth requirements, however, the unlinkable version offers much better privacy at the cost of high bandwidth consumption. While using this protocol, devices frequently change the ephemeral identifier (denoted as *EphID*) that they broadcast to other devices to avoid location tracking via broadcast identifiers. Figure 4 shows the underlying architecture of the system that works for both versions.

The protocol refers to the duration for which a device broadcasts the same EphID as an epoch. The length of an epoch is a configurable system parameter. Smartphones generate a random initial daily seed for a day and rotate it by computing the cryptographic hash using the previous seed that later generates the EphIDs. The length of the epoch can be between 10 min to 24 hours. The low-cost version disseminates a list containing the seeds of users who have reported a positive diagnosis. However, the unlinkable version hashes and stores them in a Cuckoo filter which is then distributed to other users. The Cuckoo filter is a space-efficient probabilistic data structure used to test whether an element is a member of a set [45]. The benefit of using this data structure is its query returns either “possibly in the set” or “definitely not in the set” meaning potential positives may or may not be COVID-19 infected, but negatives are certainly not. It helps to prevent identifying someone with certainty.

A hybrid design combining ideas from both versions is also available. In this design, phones generate random seeds for each time window, such as one hour, and use these seeds similar to the low-cost design to generate ephemeral identifiers for all epochs within that time window. Users upload seeds only if they are relevant to exposure estimation by other users. Depending on the length of the time window, this design offers much better protection against linking ephemeral identifiers of positive COVID-19 users than the low-cost design and enables a user to redact time windows. The protection against tracking is weaker than the unlinkable design, but this scheme has a smaller bandwidth requirement.

The proposed protocol works decentrally but utilises a backend server that shares anonymous contact information with the app running on each smartphone. This backend server is trusted for not adding or removing information shared by the users; however, remains untrusted with regards to collecting and processing personal data. The protocol reveals minimal information to the backend server where smartphones locally generate frequently changing EphIDs and broadcast them via Bluetooth Low Energy (BLE) communications. Smartphones within the range observe these EphIDs and store them together with the observed period.

If patients get diagnosed with COVID-19, healthcare authorities authorise them to publish information that aids in proximity tracing. This information contains a compact representation of their EphIDs for the infectious period that goes to the backend server. Other smartphones periodically query the backend for this information and reconstruct the corresponding EphIDs of infected patients locally. Having a record of any of these infected EphIDs stored on a smartphone indicates the corresponding user has been in contact with an infected person and the smartphone computes the owner’s risk score. If this score is above a threshold, the smartphone initiates a notification process. In this protocol, despite having a backend server, users’ privacy does not depend on it, and the privacy remains intact in the event of any compromise.

### E. PEPP-PT

**FIGURE 5.**
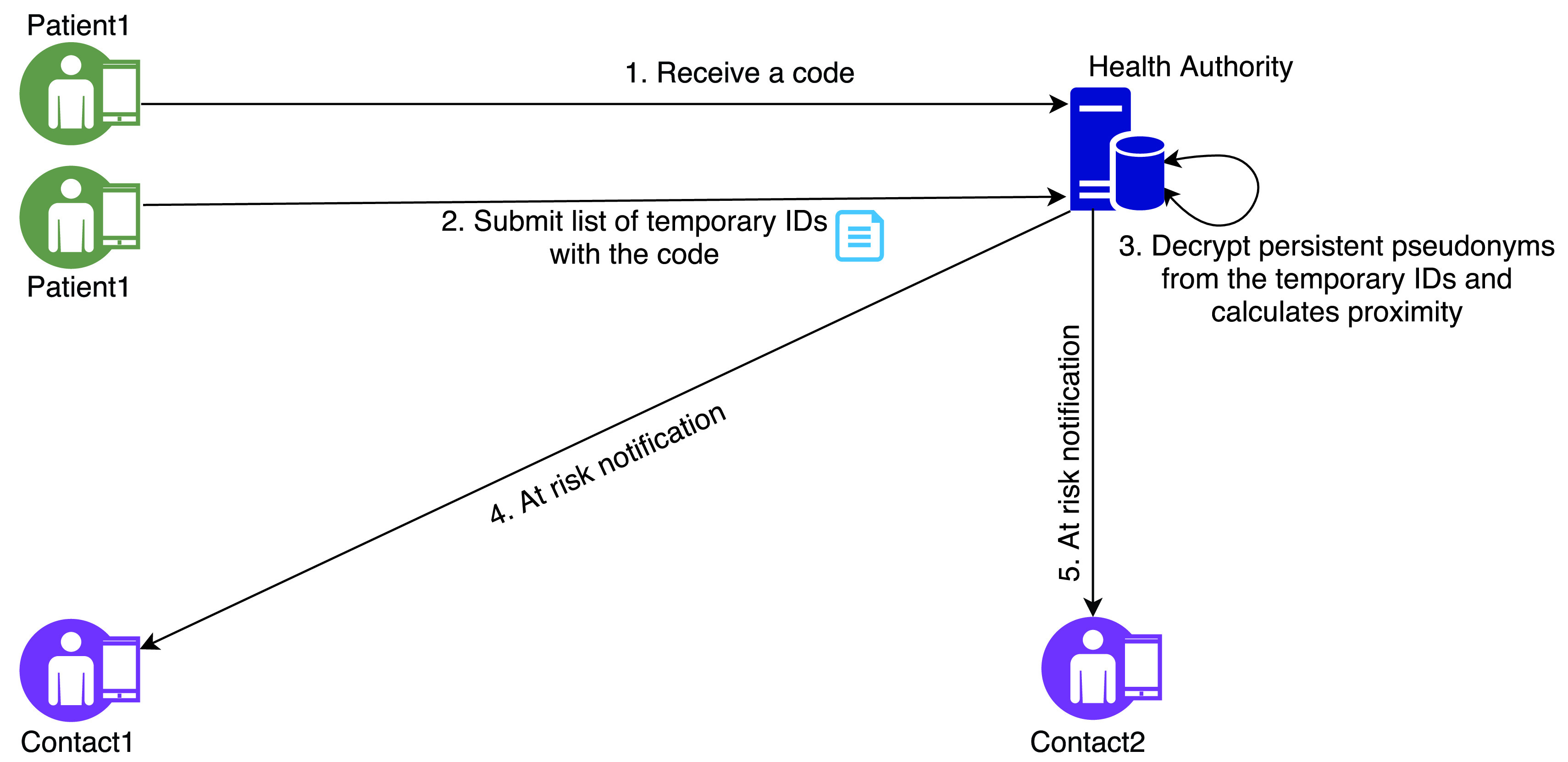
PEPP-PT protocol.

Authors in [46] have proposed the Pan-European Privacy-Preserving Proximity Tracing (PEPP-PT) which is a privacy-preserving proximity tracing system (Figure 5). PEPP-PT uses Bluetooth Low Energy (BLE) technology, allowing to notify people at risk with a 90% true positive and 10% false-negative rate. The protocol has two actors: users and healthcare officials with a server. It is assumed that PEPP-PT protocol will be utilised by a mobile app equipped with an encryption key and a persistent pseudonym. The type of encryption key and how the persistent pseudonym is generated have not been clearly outlined in the proposal. However, we assume that the key must be a public key of the server and the persistent pseudonym is generated by interacting with the server when the app is loaded for the first time.

Once the app is installed in the mobile device, it will start generating and transmitting a time-specific pseudo-random temporary ID. This temporary ID also includes the encrypted persistent pseudonym which can only be decrypted by the server. While running in the background, this app captures the signals of other BLE devices that have the app installed and exchanges temporary IDs with each other. Each app in a mobile device then continues to keep a list of their temporary IDs, each representing a contact. For each contact, the system determines the duration and the distance between the devices based on the signal output power sent by the transmitting device.

The app stores such temporary IDs in the respective device. However, once a user is tested positive for COVID-19, the healthcare official provides a specific authentication code which is used to submit the list of temporary IDs by the app. Once the server obtains data from the app of the infected person, the server accumulates the risk of contagion for each temporary ID by calculating the physical proximity and the duration with the infected users in the past. The server also decrypts the persistent pseudonym for each temporary ID and uses the pseudonym to contact the users most at risk.

### F. CAUDHT

**FIGURE 6.**
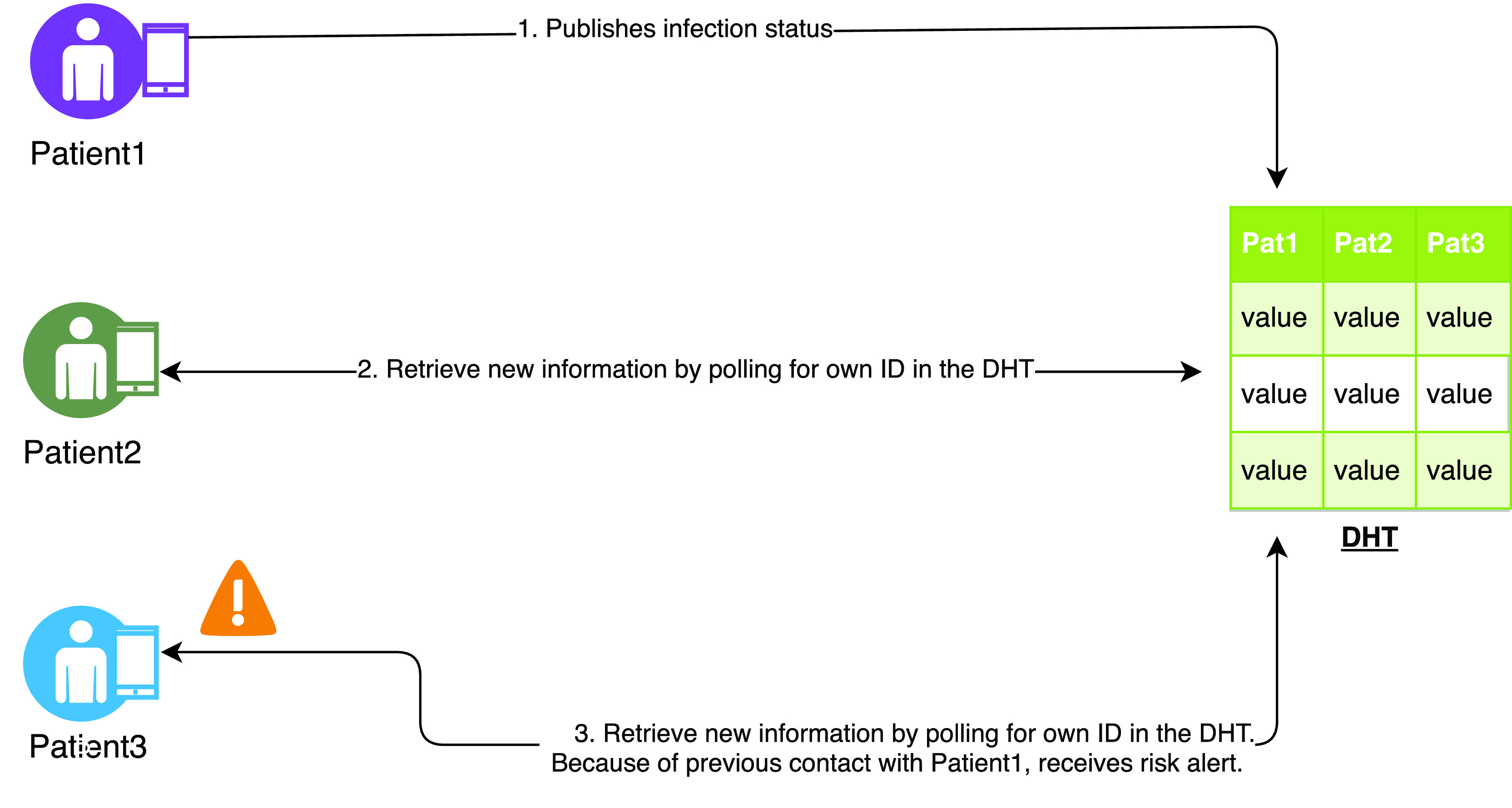
CAUDHT protocol.

Brack *et al.* have proposed CAUDHT which is a decentralised peer-to-peer system for contact tracing [47]. It utilises a distributed hash table to build a decentralised messaging system for infected patients and their contacts as shown in Figure 6. By using blind signatures, the system ensures that messages about infections are authentic and unchanged. The authors have argued that systems using ephemeral Bluetooth IDs that change every few hours for identifications also leak information. A malicious HA (Health Authority) or an attacker gaining access to the HA’s collected data would be capable of deriving some information from the transmitted contacts by correlating IDs reported by several infected patients and will be able to narrow down social or local interconnections. Therefore, they limit the HA’s responsibility to confirming the results of positively tested individuals and minimise the amount of data a centralised actor can derive from the system.

CAUDHT is decentralised by distributing works between the users of the contact tracing system using peer-to-peer technology. The system consists of several mechanisms, including a contact collection mechanism that runs continuously on every end-device and collects IDs of contacts using BLE. Following a positive test, one can announce the infection status to the system using a publication mechanism. In doing so, the user must retrieve signatures for seen IDs from the HA and publishes messages for the respective user at the corresponding location in the distributed database. An infected patient interested in retrieving a signature makes the ID blinded and sends the value to the HA. The HA signs it without learning the ID. The signature of the blinded ID is then returned, which can be unblinded only by the person who sent it — in this case, the infected patient.

The system monitors the surroundings for other users and collects IDs using the BLE. These IDs are generated from an asymmetric key pair. The secret key stays on the device while the public key ${pk}_{u}$ is used as BLE ID and broadcast to everyone nearby. Other users close by record the ${pk}_{u}$ and store it as a contact in their local history. Simultaneously, the system collects a set of public keys that later uses it to verify that contact with an infected person has indeed occurred.

### G. QUEST

Gupta *et al.* have proposed QUEST, a protocol that empowers organisations to observe individuals and implements policies for social distancing and contact tracing using WiFi connectivity data in a passive and privacy-preserving manner [48]. It has three different purposes: i) location tracing, ii) user tracing and iii) social distancing.

**FIGURE 7.**
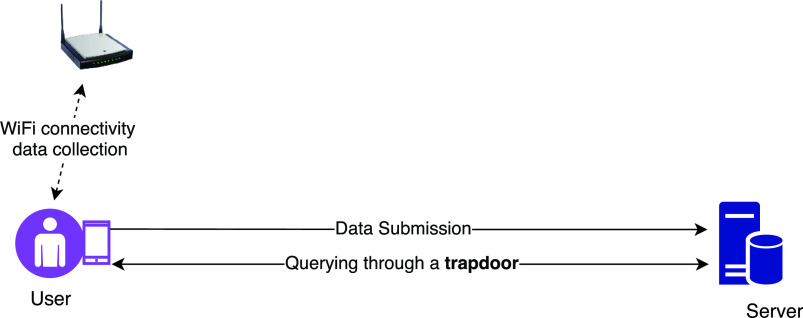
QUEST protocol.

The QUEST functionalities in this protocol determine all places that a person visited in the past 14 days. For securing the data in a privacy-preserving way, a set of three functionalities form the basis of the QUEST protocol as shown in Figure 7. The first functionality is the data collector that collects individuals’ data from the WiFi connectivity when a device connects to an access point via several network management protocols, including SNMP (Simple Network Management Protocol), NETCONF (Network Configuration) and Syslog (System Logging) protocols. Encrypter is the second functionality; it collects data for a fixed interval and implements a cryptographic technique based on the desired security level and outputs the secured data outsourced to the servers. Finally, the third functionality, the trapdoor generator, generates the secure trapdoor using two algorithms proposed by the author for query execution on secured data. For contact tracing, it confirms the submitted device-id as the real device-id of an infected person from the publisher. The trapdoors aide servers to execute queries and send back encrypted results followed by decrypting it before producing the final answer. Once the protocol obtains the results, the organisation may alert the infected users using emails or phones if they provide such consents at the time of registration. If it is not for an infected person, the results still can determine if employees are maintaining social distancing at workplaces and where they have been roaming around.

### H. PACT

Chan *et al.* [49] have proposed privacy-sensitive protocols and mechanisms for mobile contact tracing using Bluetooth technology. Before presented their protocol, they have discussed about different types of security attacks related to contact tracing apps. The types of attacks they have identified are integrity attacks, inferential attacks, reply attack and physical attack.

**FIGURE 8.**
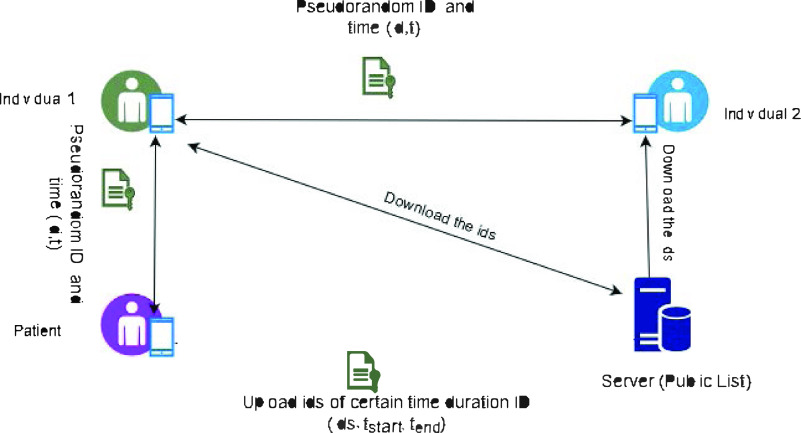
PACT protocol.

In their approach, individuals will exchange user generated seeds, pseudo-random IDs, and the time (*id, t*) when they will come to the proximity of another person and remain close for a pre-determined minimum period of time. If anyone tested positive she will voluntarily upload her user generated seeds to the public list. Other users can download the seed to check if they have been in the close proximity of these seeds. The main limitation of this approach is that they have not discussed how the public list will be maintained and who will be responsible for the governance of these public data. Figure 8 has shown the interactions among different actors in this protocol.

### I. BlueTrace

**FIGURE 9.**
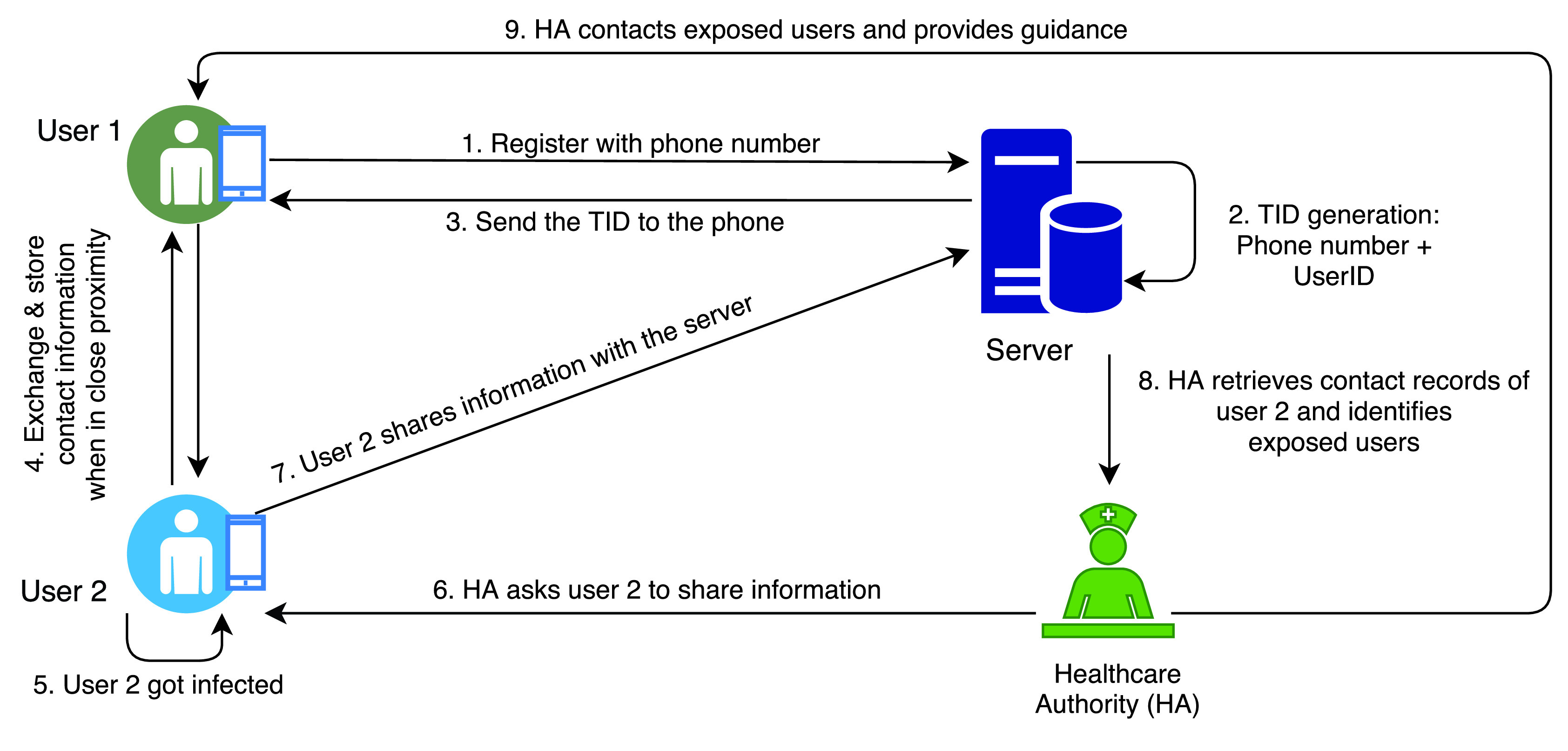
BlueTrace protocol.

The authors in [50] have proposed a privacy preserving centralised protocol for community driven contact tracing, BlueTrace, which is widely adopted or adapted by many countries. In BlueTrace protocol, two participating devices log their Bluetooth encounter information without revealing the users’ personal data. First, the user registers with a centralised server (normally the Health Authority) by providing their phone number, and then the server generates a unique, randomised *UserID* and binds this ID with the user’s phone number as shown in Figure 9. Then, the user receives temporary IDs (*TempIDs*) from the server that comprise of both encrypted (USerID, created time, and expiry time) and unencrypted (Initial Vector, Authentication Tag) fields. These TempIDS have a short lifetime of 15 minutes and the user devices are supplied with batches of forward dated TempIDs to avoid unstable Internet connection issues. When two BlueTrace devices come in close proximity, they exchange TempIDs with each other over the BLE protocol. This information includes TempID, device Model, RSSI value, organisation code, and version number of the BlueTrace protocol and is stored locally in the devices for a certain period of time (21 days in case of OpenTrace [51], an open source implementation of BlueTrace) before automatic deletion. Once a patient is confirmed to be affected and if the patient is using the app, health authorities ask the patient to upload her encounter history on the centralised server. Then the server retrieves the encounter history by decrypting the contact records. It verifies the timestamp for each TempID to find the close contacts based on the duration of exposure, distance and associated risk. After that, the health department contacts individuals who have a high likelihood of exposure to the infected person and provides appropriate guidance.

BlueTrace is vulnerable to replay and relay attacks since the protocol relies on the exchange of messages through the BLE technology. However, the authors claim that the attack vector is minimised as the TempIDs become invalid after every 15 minutes. It should be noted that the protocol stores a big pool of TempIDs in the local storage in plain text to make the IDs available when Internet connection is unstable. If a mobile device is compromised, the attackers can access and use these IDs for malicious purposes. Furthermore, the use of a centralised server in BlueTrace may also lead to a number of security risks such as identifying a targeted infected individual, tracing a target user through access to a central server and risk of data breaches, data leaks and DoS attacks.

### J. Whisper

**FIGURE 10.**
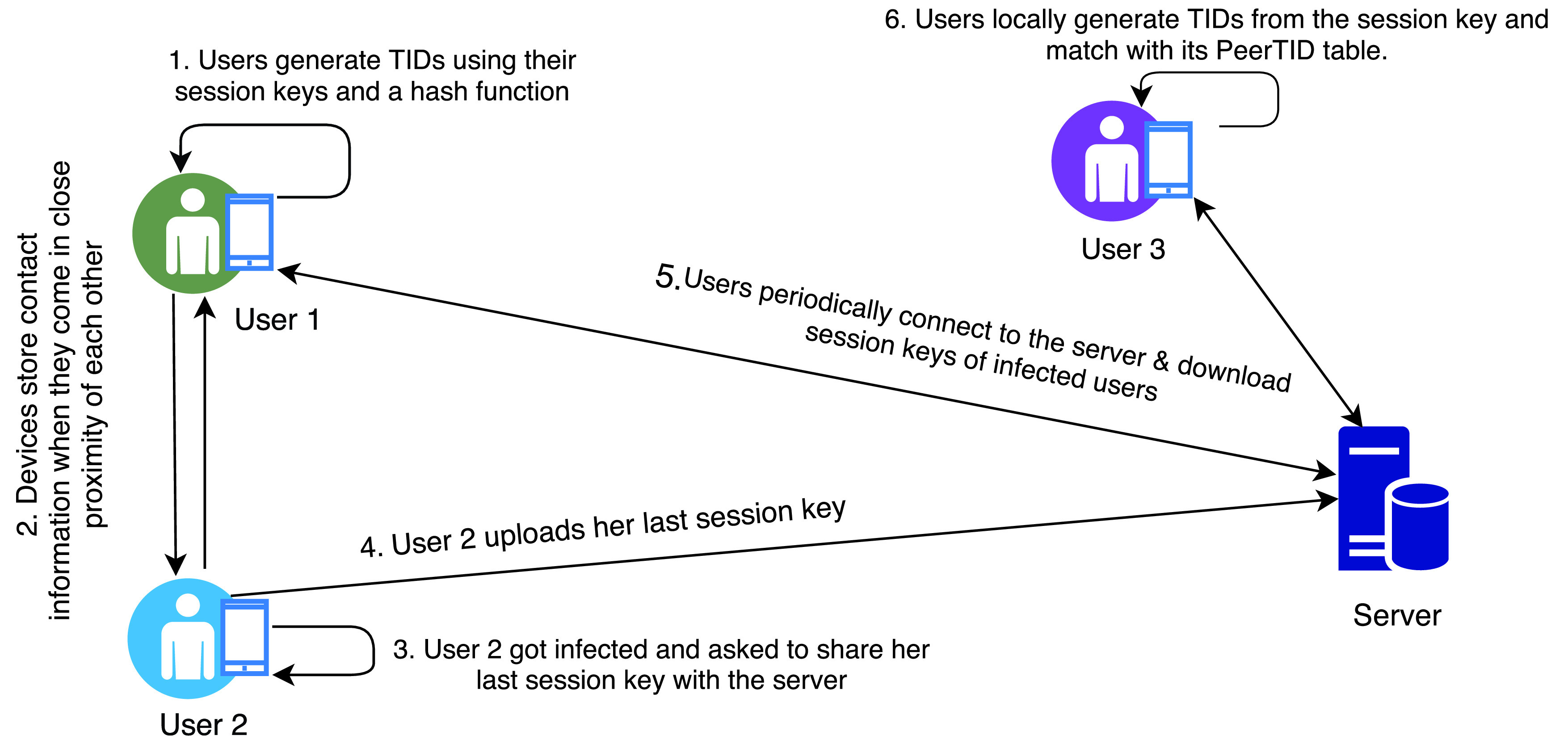
Whisper protocol.

Similar to BlueTrace, the Whisper tracing protocol also uses BLE to exchange locally generated anonymous and Temporary secure Identities (TIDs) [52]. One key difference is that the Whisper protocol uses session keys to generate TIDs and identifies individuals who have been in close contact of a confirmed patient. The protocol periodically generates pseudo-random temporary IDs using a hash function with the following input parameters: secret key ${S}$, and a counter ${C}$. A new session key is generated every week and this key is used to create TIDs on an hourly basis. When two devices make a pair, the protocol stores all encounter information in the device storage. The information is organised in a database which consists of a number of tables: i) PeerTID table (TID, timestamp), ii) Ping table (hash of peripheral address), iii) Contact table (TID, timestamp, authentication MAC), iv) SessionKey table (session keys of all peers and own), v) Join table, to map the relationship between PeerTID and SessionKey tables, vi) Scan table, to track the scan start/stop events. If a user is tested positive and has agreed to upload her contact history, she needs to share the last session key with a centralised server. Upon receiving the session key, the central server adds a description to the corresponding session key and makes it available for download. In this protocol, every Whisper node has to periodically connect to the central server for new session keys. Once new keys are available, the nodes can locally generate all TIDs and check whether the local device has been in close proximity of an infected user. Figure 10 presents an overview of the activity sequences in whisper protocol.

The whisper protocol uses a challenge-response mechanism to defend against replay and relay attacks. In this mechanism, a message authentication code is generated using a hash function and the current session key. However, if the session key is revealed, an attacker can generate the TIDs and authenticate herself as a legitimate user and also can share the session key with the server as an infected user. This could have severe impact, for an example, if the victim is a health worker, a large number of people have to be asked to go for testing or self-quarantine.

### K. The EPIC Protocol

**FIGURE 11.**
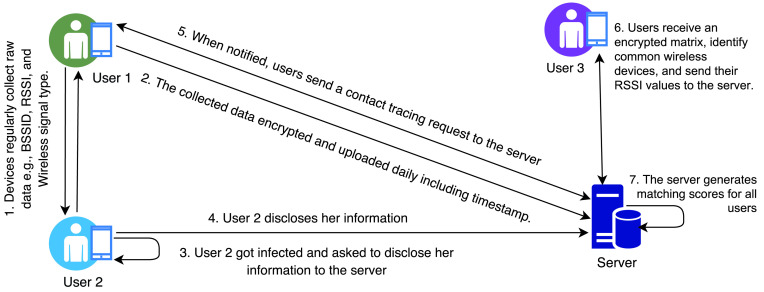
EPIC protocol.

The efficient privacy-preserving contact tracing for infection detection (EPIC) uses a weight-based matching method to determine and represent the result of the contact tracing [53]. The protocol uses wireless signals like WiFi and Bluetooth to collect the required data such as Basic Service Set Identifiers (BSSID) of wireless devices, RSSI, and wireless signal type. A smartphone regularly collects raw data about nearby WiFi and Bluetooth signals and then encrypts the data before uploading to a server. The encrypted data is uploaded to the server once a day including the timestamp of each network scan in plain text. Once a user is identified as an infected user, she has to disclose her information to the server in order to enable the server to calculate matching scores with other users. When the users are notified about the incident by the server, each regular user sends a contact tracing request including her public key. The server first matches the timestamps, then the interval, and finally common wireless devices using a privacy-preserving mechanism. The privacy-preserving mechanism implements homomorphic encryption to generate a matrix that includes encrypted subtraction results for all records between the infected user and the regular users. When a user receives the matrix, she decrypts all results and returns a binary array including 0 and 1, where 0 indicates a match for two wireless devices and vice-versa. For matched wireless devices, the user also sends RSSI values in plain text. Using the matrix and RSSI values, the server calculates matching scores for different timestamps the regular user came in contact with the infected user and sends back the encrypted scores to the users as illustrated in Figure 11.

The major issue with the EPIC protocol is that it uploads the timestamps of each network scan to the server in plain text. In addition, the RSSI values are also sent in plain text for all matched wireless devices. Thus it is vulnerable to data manipulation attack that can happen during data transmission as well as storage in the central server.

### L. Recover Protocol

The Recover protocol implements a centralised model where all devices running the protocol need to authenticate themselves on the network [54]. In this authentication process, the devices first generate a 128 bit random ID (UUID) at regular intervals and transmit it to the environment through BLE technology. Then, a remote claim procedure takes place to check whether the atomicity and non-repeatability properties are satisfied. If the ID is unique, a temporary authentication token is sent to the corresponding device to allow access to other services on the network for a certain period of time. During the contact tracing phase, mobile devices perform two operations, namely advertising and tracking. When a device detects a beacon from another Bluetooth enabled device, it starts calculating the distance and the exposure time. At regular intervals, this information (UUID-D1, UUID-D2, Contact duration, instantaneous and average distance of the contact) is sent to the Recover server to make them available for health authorities.

The Recover protocol periodically sends information to the central server. However, it is not clear whether the information is encrypted. If not, the protocol is subjected to data manipulation attack. Since the protocol implements an authentication process, it is not susceptible to replay and relay attacks. However, the security issues related to Bluetooth technology and central server are also key concerns in Recover as the protocol uses BLE technology and a central server to exchange and store information respectively.

### M. Evaluation Taxonomy

**FIGURE 12.**
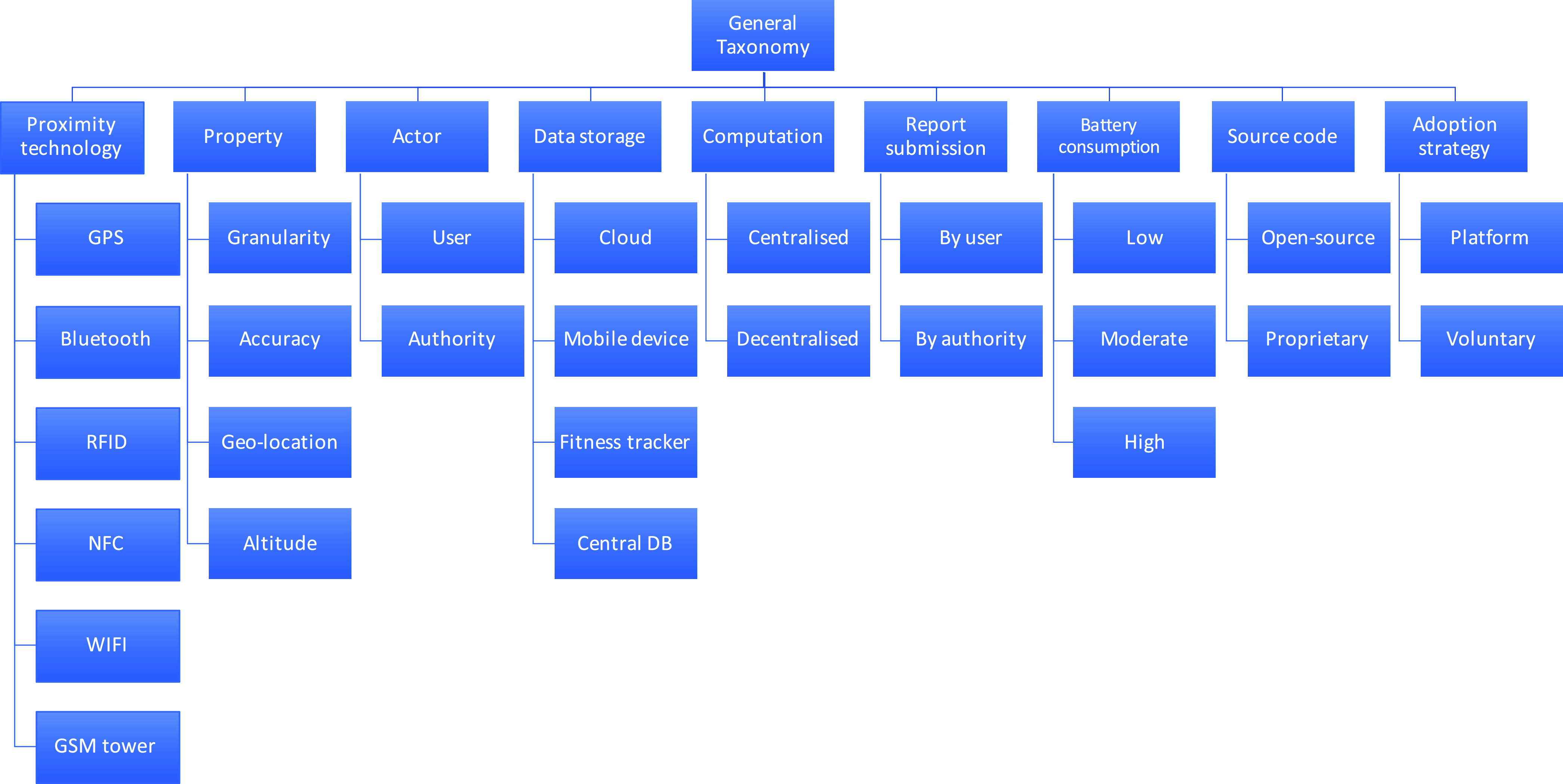
Taxonomy of general evaluation matrices.

**FIGURE 13.**
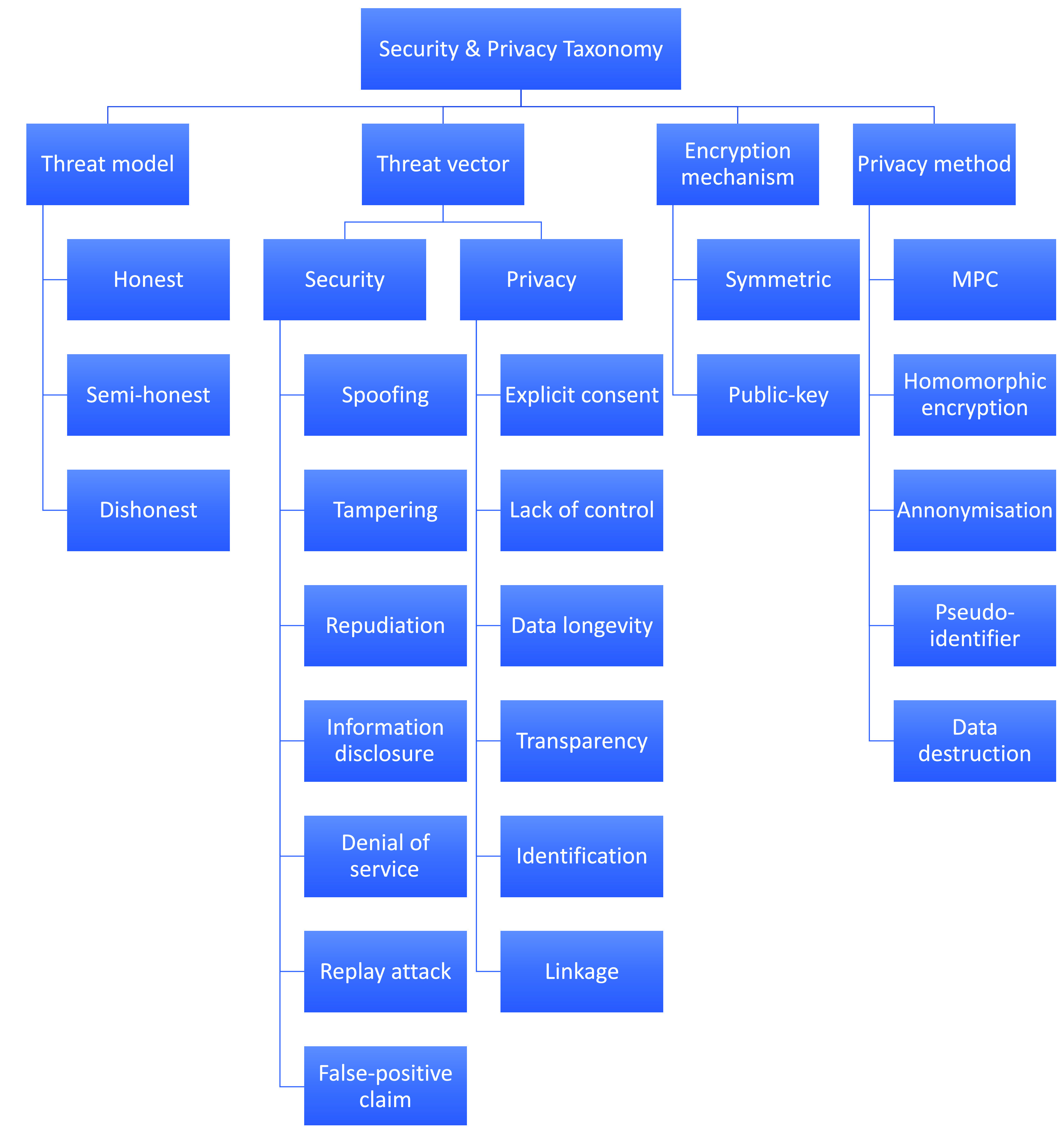
Taxonomy of security and privacy evaluation matrices.

In order to review and evaluate different existing proposals and applications for COVID-19 contact tracing, we propose taxonomies of properties. The taxonomies and their properties have been compiled based on our discussion of the proximity technologies and analysis of different protocols. Indeed, these properties will be used as evaluation matrices to compare and contrast the selected applications and proposals. The evaluation matrices presented in two different taxonomies: general taxonomy (presented in Figure 12) and security and privacy taxonomy (proposed in Figure 13). We explore different aspects of these two taxonomies next.

As illustrated in Figure 12, the taxonomy begins with the type of technologies which can be used by a protocol to detect proximity as well as to exchange data with different entities. A protocol can use GPS, Bluetooth, RFID, NFC, WiFi or even GSM mobile towers as their proximity detection technology. Each of these technologies have different properties such as granularity, accuracy, coverage, geo-location and altitude. These network technologies along with their properties are explored in Section II. Each protocol deals with different type of actors. In general, there are two types of actors: users and the authority. However, many systems differentiate between different users: a COVID-19 patient and her contacts. It is to be noted that some protocols also introduce additional actors.

Data storage category in the taxonomy signifies where the data for the system following the protocol will be stored. The system can store the data in the cloud, or a central DB (database), in the respective smart device or even in a fitness tracker. On the other hand, the computation taxonomy indicates if the required computation will be carried out in the corresponding mobile device (*the decentralised approach*) or in a remote machine controlled by the authority or a third party (a private entity) working collaboratively with the authority (*the centralised approach*). Interestingly, there is another notion of a decentralised approach in which the computation is carried out in a remote machine in a privacy-preserving manner so that the remote machine has no way of inferring any knowledge from the data [40]. Once a user is diagnosed as a COVID-19 patient, they can update their status either by themselves or it is done, on their behalf, by an authority.

Every contact tracing protocol is practically distributed using a respective mobile app running within a mobile device. Therefore, it is important to analyse the battery consumption of these protocols. Some of these apps are open-source while others are proprietary.

The successful adoption of these mobile apps will largely depend on a number of factors. Our proposed taxonomy considers a few important factors which are discussed next. In most countries, which have released contact tracing apps, the installation of such apps are voluntary in nature, whereas, it is mandatory in some countries. These mobile apps have been targeted for two major mobile platforms: Android and iOS – which account for over 99% of users.

In Figure 13, we have presented different security and privacy characteristics involving contact tracing. These issues are investigated next. There are several threat models in practice. Of them, we consider three models: honest/trusted, semi-honest and dishonest. An honest actor is trusted to hold sensitive data, to follow the protocol and carry out required computation over the data and not to exploit such data intentionally. On the other hand, a semi-honest (also known as honest-but-curious) actor is assumed to follow the protocol rules, however, many seek to gain additional information during the protocol execution. This means that even the actor follows the protocol, such an actor should not be trusted to hold sensitive data as he/she can exploit such data to infer additional information. Finally, a dishonest actor can deviate from the protocol rules and is not trusted to hold sensitive data as they can exploit such data intentionally.

Contact tracing is a rather-privacy invasive procedure as it collects sensitive personal information. Without ensuring the security and privacy of the collected data, the privacy of users can be seriously undermined. Therefore, there should be a detailed examination of the security and privacy implications of any contact tracing protocol. Towards this aim, we have created a category of threat vectors outlining different security and privacy threats.

To model the security threat vectors, we have chosen a well established threat model called STRIDE [55] developed by Microsoft. The STRIDE model with the first five threats is briefly presented below.

• **T1-Spoofing Identity:** The act of spoofing refers to a malicious user using the identity of another user (e.g. contacts). In this article, we have considered identity theft as a security threat where an attacker can spoof the identity of a device to present herself as a legitimate user to the server.
• **T2-Tampering with Data:** This threat enables a user to maliciously tamper recorded or exchanged data. There could be many such data, for example, timestamps, RSSI values, GPS location and tokens with respect to contact tracing.
• **T3-Repudiation:** This threat implies a user can repudiate certain actions, e.g. tamper certain data or exchange false data and then deny doing so.
• **T4-Information Disclosure:** This threat signifies the scenario where private or sensitive data stored in a device or central storage may be leaked to another user.
• **T5-Denial of Service:** The system that is used for computation or storage in the protocol can be the target of a denial of service attack.

The last threat of the STRIDE model is *Elevation of Privilege* which has been excluded from our consideration for this paper. This is because such a threat is more relevant for enterprise systems and has less implications in the contact tracing protocol itself. In addition to these security threats, we consider two additional threats:

• **T6-Replay attack:** This threat enables a malicious user to submit the same data more than once with the aim to maliciously impact the protocol execution.
• **T7-False-positive (FP) claim:** A user may claim of being diagnosed with COVID-19 even though they are not.

Finally, we consider the following privacy threats:

• **T8-Explicit consent:** Data regarding a user is submitted to the authority without their explicit consent.
• **T9-Lack of control:** A user has no or little control regarding how their data is shared with other entities.
• **T10-Data longevity:** If the data is stored for a longer period, it increases the chance of data abuse and decreases its security. The suggested isolation period for anyone after getting in contact with a COVID-19 patient is 21 days [56]. In line with this, we assume a data longevity period of 21 days to be privacy-friendly for any contact tracing protocol.
• **T11-Identification:** Identification is the threat of identifying an entity from a set of collected data, e.g. in our case, contact tracing data.
• **T12-Linkage:** Linkage is the most treacherous privacy threat in a contact tracing protocol in which it is attempted to match different data sets in such a way that the privacy of a certain user is undermined. Such an attack can be of two types [40]:
  • **Linkage by the authority:** The authority might try to correlate different data sets as received by different users and then re-identify the contact history of the respective users. One might argue that the whole idea of contact tracing is to enable the authorities identify the close contacts of a COVID-19 patient effectively so that the contacts can be identified, isolated and other appropriate measures can be taken. However, there are reports of COVID-19 patients being stigmatised, particularly in a few developing countries [57]–[59]. If there are malicious actors within the authority, this linkage of a COVID-19 patient with their contacts might facilitate different exploitation such as extortion by those actors. Therefore, a privacy-friendly approach would be to let the contacts know that they have been in contact with a COVID-19 patient so that they can take appropriate actions.
  • **Linkage by a user:** Contact tracing inherently enables a user to link their contacts, at least who they know and/or have been in contact for a certain amount of time. Let us consider a hypothetical scenario when a user named Alice went out of her house once in 14 days and met just one another unknown user Bob. If a contact tracing application confirms Alice that she has been in contact with a COVID-19 patient within a week, she can deduce that the patient might be Bob. The implication of this correlation would be aggravated if Alice is equipped with a life-logging device. Life-logging devices equip users to take photos automatically as they roam around. Therefore, Alice can just download the respective life-log containing Bob’s image and try re-identify him using image search online services [60] which would seriously undermine the privacy of Bob. Other practical threats might arise if a user somehow can find about other user’s contacts and/or their infection status.

To mitigate these security threats, a protocol might employ different encryption mechanisms: which can be either symmetric or public-key encryption. Similarly, there are many privacy-preserving methods which leverage Multi-party computation (MPC) [61], Homomorphic encryption [62] and other data anonymisation methods [63], [64]. A general approach to tackle the identification threat is to employ pseudo-identifiers in the collected data in such a way that other entities cannot identify the respective entity. Similarly, the collected data can be destructed after a certain period to mitigate the data longevity threat.

We would like to highlight an aspect related to the scope of this paper here. The taxonomies and the corresponding evaluation matrices that we have compiled are primarily technical in nature, focusing on the technology, process, security and privacy protection aspects. One might argue that contact tracing is a complex solution having a number of other non-technical aspects such as management, feasibility, effectiveness, accuracy and timeliness. We would like to emphasise that these other aspects, albeit very important, are dependant on some other external factors such as protocol adoption, privacy attitude, technical capability, the adoption rate of the contact tracing application and data management policies of a particular country. That is why they have not been considered as part of the taxonomies.

### N. Summary

The summary of our protocol analysis for general matrices and security and privacy matrices is presented in Section III-N1 (in Table 2) and Section III-N2 (Table 3) respectively. In those tables, we have used the symbol “ $\bullet $” to denote a certain property is satisfied by the respective protocol whereas the symbol “ $\bigcirc $” is used to denote that the respective property is not fulfilled by the protocol. Besides, the symbol “◑” is used to signify that a certain property is partially fulfilled or that the property is casually mentioned in the protocol without any further details. Finally, we use the symbol “ $\boxtimes $” to indicate that a certain property is not applicable for the respective protocol.

**TABLE 1:** Comparing Different Proximity Technologies

Technology	Location				Energy Consumption	Privacy
	Granularity	Range	Geolocation	Altitude		
GPS	Typically 5m	Worldwide	Absolute	Can Detect	High	Low
Bluetooth	NA	10m – 100m	NA	Partially Detect	Low	High
RFID	NA	1 cm – 25m	Relative	Cannot Detect	Low	High
Wi-Fi	NA	50m	Relative	Cannot Detect	Moderate	High
NFC	NA	4cm –10cm	Relative	Cannot detect	Low	High
GSM Location	50m –250m	5km – 70km	Relative	Cannot Detect	Medium	Low

**TABLE 2:** Comparison of Protocols for Different Properties With Key Symbols

Name	Network	Actor	Registration	Data Storage	Computation	Report Submission	Battery Consumption
Epione	Bluetooth	Users, healthcare provider & server	•	In devices	Decentralised	By authority	Low
TCN	Bluetooth	Reporters (patients), receivers (contacts) & Server (authority)	○	In devices & Server	Decentralised	By reporters (users)	High
Reichert et al.	GPS	Users & health authority (Server)	○	In devices & Server	Decentralised	By authority	High
DP3T	Bluetooth	Users & health authority (Server)	○	In devices & Server	Decentralised	By users	High
QUEST	WiFi	Users, hospitals & organisations	•	In devices & Server	Centralised	By authority	Low
CAUDHT	Bluetooth	Users & health authority (Server)	○	In devices & Server	Decentralised	By users	High
PEPP-PT	Bluetooth	Users & health authority (Server)	○	In devices & Server	Centralised	By authority	Low
PACT	Bluetooth	Users & health authority(server)	○	In device & server	Decentralised	By users	High
BlueTrace	Bluetooth	Users & health authority (Server)	•	In devices	Centralised	By authority	Low
Whisper	Bluetooth	Users & server	○	In devices	Decentralised	By users	High
EPIC	Bluetooth & WiFi	Users & server	○	Server	Centralised	By users	Moderate
Recover	Bluetooth & GPS (optional)	Users & health authority (server)	•	Server	Centralised	By authority	Low

•: Property satisfied ○: Property unsatisfied ◑: Property partially satisfied

**TABLE 3:** Comparison of Protocols for Security and Privacy Properties With Key Symbols and Terms

Name	Threat Model	Security Threats							Encryption Mechanism	Privacy Threats						Privacy Mechanism
		T1	T2	T3	T4	T5	T6	T7		T8	T9	T10	T11	T12 (Linkage)		
														By Authority	By Contact	
Epione	Semi-honest	•	○	○	•	○	•	•	Public key	•	•	○	•	◑	•	MPC (PSI-CA), pseudo-identifier
TCN	Semi-honest	○	•	○	○	○	•	○	$\boxtimes $	•	•	○	•	•	•	Pseudo-identifier
Reichert et al.	Semi-honest	○	○	○	○	○	○	•	$\boxtimes $	○	○	○	•	○	•	MPC
DP3T	Semi-honest	○	○	○	○	○	◑	•	Symmetric	•	•	○	•	•	•	Pseudo-identifier
PACT	Semi-honest	○	○	○	•	○	○	○	$\boxtimes $	•	◑	○	•	•	◑	Pseudo-identifier
QUEST	Honest	•	○	○	•	○	◑	○	Symmetric	•	•	○	○	○	○	Homomorphic encryption
CAUDHT	Semi-honest	○	○	○	•	○	◑	•	Public key	•	•	○	•	•	○	Blind signature
PEPP-PT	Honest	○	○	○	•	○	○	•	Public key (assumed)	•	○	•	○	○	○	Pseudo-identifier
BlueTrace	Honest	•	•	•	•	○	◑	•	Symmetric	•	•	•	○	○	•	Pseudo-identifier
Whisper	Semi-honest	○	•	•	○	○	•	○	$\boxtimes $	•	•	○	•	•	•	Pseudo-identifier
EPIC	Semi-honest	○	○	○	◑	○	○	○	Public key	•	◑	○	•	•	•	Homomorphic encryption
Recover	Honest	•	○	○	○	○	•	•	$\boxtimes $	○	○	○	○	○	•	Pseudo-identifier

T1: Spoofing identity T2: Tampering with data T3: Repudiation T4: Information disclosure

T5: Denial of service T6: Replay attack T7: False-positive claim T8: Explicit consent

T9: Lack of control T10: Data longevity T11: Identification T12: Linkage

•: Property satisfied ○: Property unsatisfied ◑:Property partially satisfied $\boxtimes $: Not applicable

#### 1) Evaluation of General Matrices

Following Table 2, the values in columns for a particular protocol have been taken from our analysis of the respective protocol. However, some values require additional explanation which is provided next. For example, the battery consumption (the last column in Table 2) is considered low for Epione as the computation is carried out in the Epione server in a privacy-preserving decentralised fashion which requires no heavy computation in the mobile phone of a user. For the TCN protocol, the data is stored in devices as well in the server. The computation is carried out in respective mobile devices in a decentralised fashion and hence, requiring high battery consumption. The battery consumption of the proposal of Reichert *et al.* [42] is considered high as the users need to carry out the computation, using the garbled circuit, in their own mobile device. Similarly, the battery consumption in DP3T, CAUDHT, PACT and Whisper protocols is high since all computations are performed in the local devices to identify exposed users. In contrast, QUEST, PEEP-PT, BlueTrace and Recover protocols consume low battery power since all computational tasks are carried out by the server system. Although EPIC implements a central server that performs most of the computations for contact tracing, mobile devices also need to execute certain operations on encrypted data and send the outputs to the server system in this process. Therefore, the protocol results in moderate battery consumption.

#### 2) Evaluation and Privacy Matrices

Similarly, Table 3 analyses the security and privacy issues for different protocols. Following the table, Epione has a semi-honest threat model for the Epione server. Threat T1 can be mitigated with a proper registration process which can distinguish between different users. The details of the registration process in Epione has not been provided in the proposal. However, we can assume such a registration process would be essential to identify a particular user and their app, so that the healthcare provider can positively identify the app when app data is submitted. There has been no discussion regarding threats T2 and T3 in Epione. The token seeds are encrypted with the public key of the Epione server and then exchanged, thus mitigating threat T4. The server itself can act as a single point of failure and can be the target of a DoS attack (T5). Even though, Epione did not consider this, we argue that Epione implicitly satisfies T6. This is because only seeds are shared with the server and tokens are restored at the server’s end and hence there is nothing to gain for an attacker to launch a replay attack. Therefore, we argue that Epione implicitly satisfies T6. Epione requires a healthcare provider to authorise if a user has been positively diagnosed, this mitigates T7 threat.

Epione requires users’ consent to share data with the server and the users have full control over their data. These ensure that threats T8 and T9 are mitigated. There is no discussion of data deletion, implying T10 not being handled. The tokens are generated using pseudo-identifiers, hence, there is no way of identifying a user from the exchanged tokens, signifying the fulfilment of T11. The usage of a strong privacy-preserving PSI-CA mechanism and pseudo-identifier tokens ensure that other contacts cannot link tokens of different users. This is true for the Epione server. However, Epione has a provider entity (modelled as part of the authority) which can link a user when they submit a token. Because of this, we have used the “◑” symbol in the authority cell.

TCN with a semi-honest model has no registration process and hence cannot mitigate T1 threat. It has a mechanism to ensure integrity and therefore can mitigate T2. However, it did not consider any encryption mechanism to mitigate T3 and T4 threats. Utilising a server in their protocol makes it vulnerable against a DoS attack which has not been considered, implying T5 not being handled. TCN has a defence mechanism against T6 along with T8 and T9. TCN did not explicitly consider how a patient will update her status of being diagnosed, signifying T7 not being handled. The usage of pseudo-identifiers as a privacy mechanism enables it to satisfy T11, however, the data longevity was not addressed, indicating T10 not being handled. The protocol defends against linkage attacks by the authority and other contacts.

The MPC proposal of Reichert *et al.* [42] also assumes a semi-honest server without any registration process (T1 not being fulfilled). Unfortunately, their proposal lacks of any encryption and other required mechanisms to mitigate T2, T3, T4, T5 and T6. However it ensures a defence against T7 by using the authority to update the status of a user. With respect to privacy threats, this proposal does not satisfy T8, T9 and T10. However, T11 is satisfied as the token does not contain any identifying data. The usage of MPC ensures a safeguard against the linkage attack by other contacts, however, the authority has full access to GPS location data of the two patients which can be used to link these two users.

DP3T belongs to the semi-honest model and it does not employ a registration process. Thus, the protocol is unable to mitigate threat T1. Since no encryption mechanism (e.g. symmetric encryption or digital signature) are used in DP3T, it cannot resist T2, T3 and T4. Similarly, there is no protection mechanism available to safeguard the system against DoS attacks (implying subject to T5). However, the short lifetime of TIDs partially mitigates the threat of replay attacks (T6) in DP3T whereas it employs a preventive mechanism to mitigate T7 threat. Regarding privacy threats, the protocol is resistant against T8, T9, T11 and T12. However, data retention time is not specified in the protocol and therefore, we assume DP3T is vulnerable to T10.

In PACT, users do not store any personal information (e.g., name, phone number) in the server, thus users do not need to trust the server with their information and can be termed as a semi-honest server. There is no registration procedure in the protocol and therefore, it is vulnerable to T1 threat. Furthermore, the protocol is also exposed to T2, T3, and T4 threats due to lack of any authentication and encryption mechanisms [65]. The users do not encrypt the pseudo-anonymous IDs and timestamp values are stored in plain text. It should be noted that the server can be a target of DoS attacks (T5) that may have severe impact on the system. We assume that threats T6 and T7 are not considered in this protocol since there is no information regarding these two threats. However, users have full consent while uploading the data to the server (which mitigates T8 threat). All information is stored locally and the matching computation is also computed in user’s mobile device, therefore it defends T9. Regarding data longevity, there is no indication of the lifetime of stored information (i.e., subject to T10 threat). Since there is no registration information stored in the server, the protocol is resilient against identification threat (T11). Finally, the use of mobile devices for data storage and computations ensure that PACT can successfully mitigate linkage attack by authority and users (T12). The contact tracing mechanism in QUEST is based on a central server and belongs to the honest threat model. It mitigates T1 threat by implementing a registration process. However, there is no indication of integrity and authenticity checking mechanisms in QUEST and therefore, we assume the protocol is subject to T2 and T3 threats. The use of encryption mechanism ensures that information disclosure (T4) threat is eradicated in QUEST. Nonetheless, DoS attacks (T5) can be launched against the central server to make it unavailable for a certain period of time. Like DP3T, the short lifetime of TIDs ensures partial mitigation of T6 threat in QUEST, however, no attempts have been made to eliminate T7 threat. Apart from these security threats, the protocol successfully defends against T8, and T9 privacy threats through the use of user consents and providing some sort of user control on data whereas T10 and T11 are not addressed as per protocol specification. Similarly, there is no protection against linkage attack (T12) in QUEST.

The threat model of CAUDHT is identified as semi-honest where the contact tracing procedure is decentralised and is run by mobile devices. According to the protocol description, it does not have any registration process, integrity and authenticity checking mechanisms and therefore, is vulnerable to T1, T2 and T3 threats. Nonetheless, the usage of encryption mechanism ensures mitigation of T4 threat whereas, like other protocols, T5 is not addressed in this protocol. Similar to QUEST, CAUDHT partially mitigates T6 and fully prevents T7 threat. In terms of privacy threats, the protocol is not susceptible to T8, T9, T11 and T12 (linkage by authority) threats. However, it is subject to T10 due to lack of a data retention policy and T12 (linkage by contact) threats due to underlying contact tracing mechanism.

BlueTrace implements a central server which is assumed an honest server. The registration process in BlueTrace mitigates T1 threat whereas the use of authentication tag and encryption mechanism safeguards the system against T2, T3, and T4 security threats. However, like other protocols, BlueTrace is also subject to DoS attacks (T5) for using a central server to generate TIDs and identify exposed users. Regarding T6, the threat of replay attack is minimal in this protocol since the TIDs have a short lifetime of 15 minutes only. One key feature of BlueTrace is that only authenticated users can send the contact history to the server and thus successfully mitigates T7. Similarly, the requirements of explicit consents of users, deletion of collected information after 21-days ensure that the protocol defends against T8, T9 and T10 privacy threats. However, it is susceptible to T11 threat as the server/authority has full access to uploaded information and the user IDs are combined with the corresponding phone number. For the same reason, there is a potential threat of linkage by authority in BlueTrace. In contrast, linkage by contact is not possible except in special situations mentioned in Section III-M.

The threat model of Whisper is identified as semi-honest since its central server only holds and shares the last session key of the infected users with others for computing the risk of exposure. The protocol does not implement any registration mechanism, indicating T1 is not addressed. However, the use of authentication MAC reduces the chance of both T2 and T3 threats. However, it is assumed that Whisper is vulnerable to T4 threat since there is no indication of using any encryption mechanisms. Thus, if a mobile device is compromised, the attacker may disclose all sensitive information. The decentralised architecture of Whisper makes it less susceptible to DoS attack (T5). However, attackers may launch a DoS attack against the server and thus prevent the users from downloading session keys of the infected users. The protocol successfully mitigates T6 threat using a challenge-response mechanism whereas it is prone to T7 threat (false-positive). Since the users need to share the last session key with the server, compromisation or manipulation of this key can trigger false-positives. T8 and T9 privacy threats are mitigated in Whisper as it requires explicit consent of users and the users have full control of their data. However, there is no information about data retention policy and therefore, we assume it is subject to T10 threat. Due to very minimal involvement of the server in tracing exposed users, the possibility of T11 threat is negligible. Similarly, it is not possible to lodge a linkage attack by the authority as they do not have any access to user data. However, like BlueTrace, the linkage by contact is only possible for special situations.

The EPIC protocol stores encrypted information and also performs contact tracing computations on encrypted data. Since the server is unable to make the linkage between a patient and contact, we classify it as a semi-honest server. There is no indication of using a registration process in EPIC and therefore, we assume that the protocol is unable to defend against T1 threat. Similarly, the protocol is vulnerable to T2, and T3 threats since no authentication mechanism is used in EPIC. Although, the protocol sends timestamps and RSSI values in plain text, sensitive and private information is encrypted and thus it partially eliminates T4 threat. Regarding T5 threat, we assume that the server can be a target of DoS attacks and it will have severe impacts on the system. We also assume that threats T6 and T7 are not considered in EPIC as per protocol specifications. However, it is clear that infectious users must be agreed to disclose their information with the server (mitigating T8) and they have some level of control on their own data (T9). There is no indication about the lifetime of stored information and therefore, EPIC is subject to T10 threat. However, the use of homomorphic encryption ensures that threat T11 is successfully mitigated in this protocol. For the same reason, the protocol is secure against linkage attack by authority and the user (T12).

In Recover, the server has full control over the users and contact information, and thus belongs to the honest threat model. From the specification, it can be assumed that the protocol implements a registration process and thus mitigates T1 threat. However, there is no explicit information to identify whether the protocol mitigates T2, T3, and T4 threats. The use of the central server also makes the protocol vulnerable to T5. Recover mitigates T6 threat by employing an authentication mechanism for device to device communication. In addition, the protocol defends against T7 threat since the health authority identifies both infected users and exposed users using the contact records stored in the server. Regarding privacy threats, the protocol is susceptible to T8, T9, T10, T11 and T12 (linkage by authority) threats. This is because the authority can identify any users and access their information without permission. There is no information available regarding data retention policy (i.e., subject to T10 threat). However, the linkage attack by contact is not possible since all information is handled by the server.

## IV. Review of Existing Contact Tracing Apps

In this section, we review a number of contact tracing applications adopted in different countries.

### A. Australia - COVIDSafe

The Australian government has launched the COVIDSafe app [66], which enables the exchange of a series of digital handshakes to identify whether two people using the app come within 1.5m proximity for at least 15 minutes.

Individuals are required to download and install the COVIDSafe app from either Apple App store or Google Play. This app needs user registration including their name (or pseudonym), age range, postcode and phone number. The collected information is encrypted and stored in the national COVIDSafe data store. However, the information can only be decrypted in the event of an app user tested positive or exposed to an infected person. The app continuously generates Bluetooth beacons (anonymised IDs) and exchanges the IDs with nearby individuals who also use the COVIDSafe app. The anonymised IDs change every two hours and are stored in encrypted forms on phones for 21 days. If someone is tested positive for COVID-19, she uses the app to provide her consents and uploads her contact records on the server. The received signal strength, phone model and other data are used to determine who needs to be contacted by health authorities. The app is voluntary in Australia and has 6 million+ downloads, over 25% of the total Australian population. It has been reported in the media that the COVIDSafe app has been successfully used by health officials to access data of a COVID-19 patient in Victoria in May, 2020 [67].

#### 1) Critical Review

One of the main critics of this app is that the data are stored by an international cloud service provider AWS (Amazon Web Service). There is an on-going legal discussion within the civil society whether the US government has subpoena power over this data. Secondly, as this is a centralised approach and the contact list of infected app users are uploaded to the server, there may be a potential risk of de-anonymisation. Apart from these, initially the iOS app failed to capture all Bluetooth handshakes from the nearby devices when it was running in the background. In addition, it was potentially interfering with another app designed for diabetic monitoring [68]. However, the Digital Transformation Authority (DTA), who is responsible for the app, released the source code to the public, and rolled out four updates within 6 weeks to enhance the security and stability of the app.

### B. China - Chinese Health Code System

The National Health Commission (NHC) of China utilises automated platforms to transmit and obtain details on the type and severity of diseases, advises people about how to avoid outbreaks and warns what to do if infected. The government uses high-speed telecommunications services to provide safe travel information to their citizens. In addition to the use of drones for surveillance, Chinese authorities have released a mobile app that tracks people and alerts them if they have been in “close contact with someone infected” using the application [35].

To measure the risk level of any particular person, China also has introduced a color-code app that uses three colors, namely, green, yellow and red. Signs displaying Quick Response (QR) codes are displayed at public checkpoints, including office buildings, shopping centres, bus and train stations, and airports. Users are required to scan the QR codes with their phones and wait for their devices to display a colour-coded signal to determine whether they can proceed. A green code allows the users unrestricted movements, while a yellow code requires seven days of quarantine. If the code returned is red, the user is determined to be either a confirmed case of COVID-19 or a close contact, and must be placed in isolation. For controlling people’s movement, the app also has contact-tracing mechanisms in place to notify users if they have come in contact with infected people. For this purpose, China uses mobile cell data to determine the close contact and stores personal information such as name, national identity card number, phone number, and home address. In addition, the app also asks questions which are relatively more invasive, querying users on health status and travel history, and requesting them to identify any close contacts diagnosed with the COVID-19 during registration.

#### 1) Critical Review

China’s city of Wuhan reported the first cluster of COVID-19 outbreak. The country used extensive measures to rapidly control the virus while it was learning about the severity, the infection mechanism and how to manage this situation. It was more or less unknown to the humanity how to contain the spread of such virus as it is a novel virus. It has used a variety of digital technologies, not just apps, to tackle the situation. In western standards, the app and technologies used by the Chinese government are privacy invasive in nature. The accuracy is also in question as estimating close contact using GSM cell location is not always precise.

### C. India - AarogyaSetu

India has rolled out its mobile contact tracing app called AarogyaSetu [69] in April. It is a multi-lingual app which is useful for users to know whether they are at the risk of getting infected with COVID-19. The app can help a user to identify possible COVID-19 ‘hotspot’ around her area. It can also help people stay safe and adopt necessary precautions in some areas where there are positive cases and accordingly, help stop or prevent community transmission to some extent.

Using geo-tagging (enabled by GSM technology), it can also alert a specific user about their proximity to a nearby infection case or hotspot. The app also helps users self-identify their risk and monitor their health conditions, considering the difficult situation where it is not particularly safe to step out and visit health clinics. If someone met a person within the last two weeks who has later tested positive, the app calculates the risk of infection based on how recent it was and level of proximity, and provides guidelines. In terms of registration information, it collects name, phone number, gender, travel history and whether someone is a smoker. The government made this app mandatory for citizens living in containment zones and for all government and private sector employees.

#### 1) Critical Review

The main critic of this app is that it collects absolute location information (e.g., geo-location using GPS). According to the developers of the app, the application fetches a user’s location during the time of registration, at the time of self-assessment, and when a user submits her contact tracing data voluntarily through the app or when it retrieves the contact tracing data of a user after confirmed as a COVID-19 patient. Though it has been claimed that the app stores encrypted location information, but there are potential risks of cyber hacking or state surveillance on the citizens. In addition, there is no clear information about who can access the information stored in data centers and how long the data will be kept.

### D. Singapore - Trace Together

Singapore’s TraceTogether [8] app is a Bluetooth-based contact tracing mechanism which uses a range of cryptographic identity protections. For instance, the app employs rotating encrypted IDs that are generated by the server. This enables the server to decrypt users’ IDs and identify exposed individuals. The app logs users’ pseudonymised IDs and contact records via the BlueTrace protocol [50] discussed in Section III. These IDs are rotated periodically, with the central service being able to map back to the corresponding phone number in case of a positive diagnosis. It should be noted that diagnoses are authenticated by a QR code to prevent false-positives. Recently, Singapore has started to use bluetooth token to incorporate senior citizen in the contact tracing regime who do not carry smartphones or do not have access to mobile phones [70].

#### 1) Critical Review

The approach followed in the TraceTogether app is more privacy aware compared to the approach used in AarogyaSetu as the app does not collect geo-location of the individuals. However, the app is mostly centralised, therefore more susceptible for certain types of cyber attacks. Government has released the source code and this has made the application well studied and replicated elsewhere. The Australian government has also used BlueTrace to develop their COVIDSafe app.

### E. South Korea - Corona 100m

South Korea has used mobile phone location data, along with the country’s prolific CCTV and credit card transaction records [71]. Authorities retrospectively track the movements of people who later test positive. Because the technology uses GPS location data, and phone companies in South Korea require all customers to provide their real names and national government registration numbers, it is nearly impossible to avoid being tracked if someone owns a smartphone. The routes taken by infected people are often published online, while an alert message is sent to the people who had visited the same locations. However, some users subject to quarantine requirements reportedly flouted tracking systems by simply leaving their phones at home. Therefore, the government asked the repeat offenders to begin wearing tracking wristbands.

#### 1) Critical Review

The South Korean app was one of the first set of apps that almost completely discounted privacy issues. It definitely violates the privacy of the individuals which is a big concern. However, it was the early days of COVID-19 and people and authorities were searching for anything that could help to save human lives. Many of these early applications were influenced by the practices and effectiveness of those in China as described in Section IV-B.

### F. UK - NHS COVID-19 App

The NHS (National Health Service) COVID-19 app [72] uses Bluetooth handshakes to register proximity events or contacts between smartphone users including factors such as duration of the ‘contact event’ and the distance between the devices. This information is fed to an NHS clinical algorithm that is being designed to estimate the level of infection risk and trigger notifications if a user subsequently experiences COVID-19 symptoms. The government is promoting the app as an essential component of its response to fight against COVID-19. Upon startup, the app requests Bluetooth and push notification permissions and reaches out to api.svc-covid19.nhs.uk with an activation code, a push notification token, and a portion of the user-entered postal code. Then the server replies with a linking-ID that gets stored in the user’s app settings.

One major component of the UK’s approach is that it has opted to create a so-called ‘centralised’ system for COVID-19 contact tracing — which leads to a number of specific challenges. While the NHS COVID-19 app stores contact events on the user’s device initially, at the point when (or if) a user chooses to report themselves having COVID-19 symptoms, then all their contact events data are uploaded to a central server. This means it is not just a user’s own identifier but a list of identifiers encountered over the past 28 days, therefore, essentially, a graph of their recent social interactions. The server then runs an algorithm to compute a risk score that is used to determine whether people who came in contact with the infected person should be notified. It has been claimed that the Android version requests location permissions (due to Android’s permission granularity, *ACCESS_ FINE_ LOCATION* in the Android app is necessary for using Bluetooth). However, regarding the iOS version, it does not request location permissions. The UK government has recently abandoned the centralised coronavirus contact tracing app. They have decided to switch to an alternative application designed by Apple and Google [73].

#### 1) Critical Review

The UK app follows a similar approach as the Australian app and uses a ‘background refresh’ feature to keep reactivating the app, plus push notifications that ask the users to manually restart it. Researchers have found that the iPhone app seems to regularly reactivate the phones provided there are other devices (including Android phones) nearby running the app.

The other major concern is that the app may violate privacy by giving the authorities data on users’ locations, which could then be stored and misused. However, it is worth noting that the UK app does not trace contacts using a phone’s location. Users are asked for the first half of their postcode as a way of helping the NHS to understand how many people are infected within a relatively wide area and plan resources accordingly.

The centralised model implies that health authorities have access to a list of devices a user has recently been in contact with. But it also avoids the necessity to broadcast an anonymised list of people who have reported symptoms. Re-identifying people from anonymised data is a valid concern. But this still needs several pieces of information about an individual to work – and at present the server does not store or see any contextual information that would be useful for re-identification.

As with all complex systems, there is a series of trade-offs to be made. But the privacy protections built into the UK’s app are somewhat robust, and the barriers to breaking them or misusing the data are high. Problems may still emerge and the app’s current version may not be as effective as expected, however, the authorities are trying to overcome the issues in future updates.

### G. Other Popular Smartphone Apps to Track COVID-19

In addition to governments, different universities (e.g., Stanford University) and commercial companies (e.g., Apple and Google) have joined in app based initiatives. Apple and Google published APIs to use the Bluetooth functionality in their respective mobile operating system [74]. It lets iOS and Android phones to communicate with each other over Bluetooth, allowing developers to build a contact tracing app that will work for both. Some countries have started using these APIs such as Austria and Estonia [75], while some others have rejected the use of this API such as France and USA [76].

Similarly, a contact tracing app, CovidWatch is developed by a pair of researchers from Stanford University and the University of Waterloo [77]. It uses Bluetooth signals to detect users when they are in close proximity to each other and alerts them anonymously if they were in contact with someone who has been tested positive. A distinguishing feature of this app is that any third party, including the government will not be able to track who was exposed to whom. It has been among the early apps to release an open-source protocol for privacy-preserving, decentralised Bluetooth based contact tracing. Table 4 shows the summary of our findings.

**TABLE 4:** Comparison of Contact Tracing Apps

Country	Name of App	Protocol	Technology	Source Code	Platform	Battery Usage	Voluntary
Australia	COVIDSafe	BlueTrace	Bluetooth	Open	Android & iOS	Low	Yes
China	Health code system	N/A	QR Code	Closed	Android & iOS	Low	No
India	Aarogya Setu	N/A	GPS	Open	Android & iOS	Moderate	No
Singapore	Trace Together	BlueTrace	Bluetooth	Open	Android & iOS	Low	Yes
South Korea	Corona 100m	N/A	GPS	Open	Android & iOS	Low	Yes
UK	NHS COVID-19 App	N/A	Bluetooth	Open	Android & iOS	Low	Yes
Global	CovidWatch	TCN	Bluetooth	Open	Android & iOS	Moderate	Yes

## V. Discussion

It is evident from the above reviews in sections II, III and IV that a handful of protocols and mobile apps have been available as the pandemic enters its tenth month since the first case. These protocols and apps, however, demonstrate significant differences in underlying technologies and approaches for managing various security and privacy traits. In this section, we highlight the key differences (Section V-A) accompanying with the security and privacy considerations followed by a series of recommendations (Section V-C) for an improved privacy-preserving COVID-19 contact tracing application.

### A. Analysis

**FIGURE 14.**
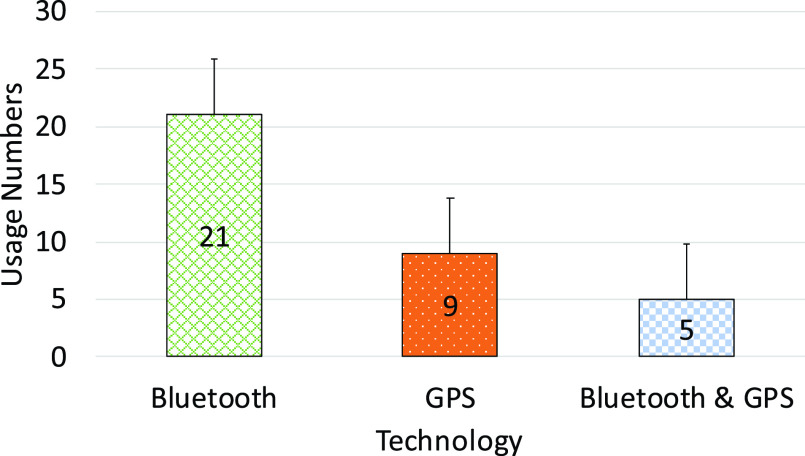
Technology used by different apps.

In Section II, we discussed various types of technologies to detect proximity among two different persons, such as GPS, Bluetooth and Wi-Fi. Among these technologies, GPS captures the absolute location of a person, making it more privacy-invasive in comparison to say Bluetooth which only estimates if two people come in close contact without recording the absolute location of the encounter. This privacy-friendly feature has made the majority of the proposed app developers to adapt Bluetooth as the underlying proximity detection network [78] (Figure 14). Furthermore, we have analysed 12 different protocols against a set of security and privacy threat vectors.

**FIGURE 15.**
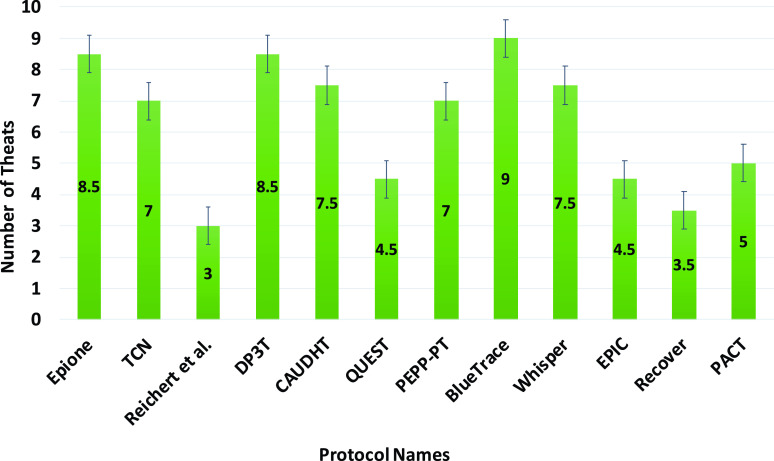
Number of threats mitigated by each protocol.

Figure 15 plots the number of alleviated threats by the selected protocols. In our analysis, we found BlueTrace is mitigating the highest number of threats (9), followed by Epione and DP3T with 8.5 each, where the fraction implies that the respective protocol does not adequately mitigate at least one threat. Among others, the MPC proposal by Reichert *et al.* has the lowest number of alleviated threats with only 3.

**FIGURE 16.**
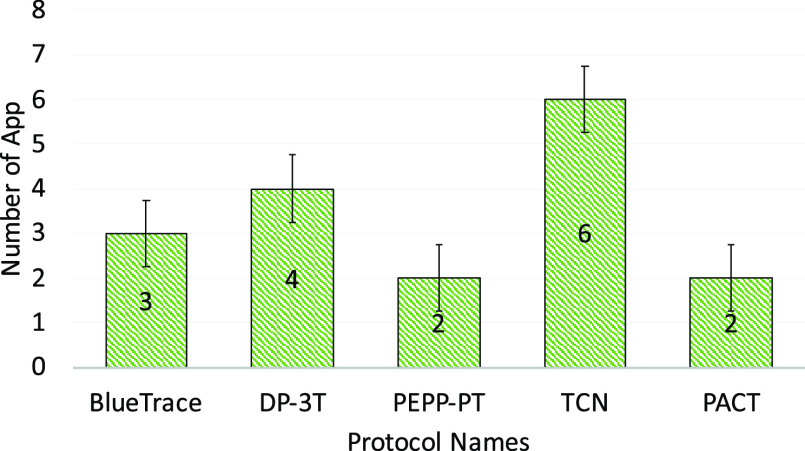
Protocol used by number of apps.

To understand which protocols have been utilised more in different apps, we collected the number of adoptions for each protocol (illustrated in Figure 16) [79]. According to our analysis, the number of protocols adopted so far is just 5 out of 12. With six apps utilising TCN, it is the most widely used protocol in the current setting of contact tracing applications. On the other hand, DP3T has been utilised by 4 apps. Other three protocols, BlueTrace, PEEP-PT and PACT, have been used by 3, 2 and 2 apps respectively.

It is to be noted that even with a moderate threat coverage of 7, TCN is the most widely used protocol as of now. Although the underlying rationale behind this adaptation rate is puzzling to assume, there are a number of other factors, *such as ease of deployment, political will, the credibility of the researchers and so on*. To wrap up, there is still no app utilising the other seven protocols.

### B. Security and Privacy Considerations

Our analysis identified some security and privacy considerations for contact tracing scenarios and are summarised below:

• **Access Control:** Access to individuals’ private information is sensitive; therefore, accessing such data needs controlling. The access should only be granted to limited health professionals. To avoid misuse the law enforcement and federal government, should never get access to such data. Sometimes legal protection is essential to build trust between the government and the citizen. Otherwise, technologies can quickly become a surveillance tool for the government. In addition to the legal framework, the technical implementation is also crucial. The need for proper authentication and authorisation is vital and must be audited from time to time.
• **Data Storage:** Design patterns should consider storing the user data in the local device as much as possible. The data should not leave the users’ device until that is absolutely necessary. For example, the tracing data (pseudo-anonymous ID) should not be uploaded to the server until someone becomes infected. Similarly, the contact tracing application must implement a data disposal mechanism to ensure that data will be automatically deleted after a certain period of time.
• **Data Management:** Contact tracing applications require a robust and interoperable data management system for linking users with confirmed and probable COVID-19 to their contacts. In some cases, the systems may also require to seamlessly integrate with other modules to facilitate more complicated epidemiological analyses. Although it is important to incorporate data security and confidentiality mechanisms into all phases related to contact tracing activities, most of the applications overlook this.
• **Data Transmission:** Most of the contact tracing applications require data transmission between mobile devices and a central server to register new users, upload encounter information, notify exposed users and so on. It has been noticed that most of the applications implement cryptographic schemes to secure only those steps that involve transmission of sensitive information whereas some are not using transport layer security (TLS) at all. For example, a recent investigation by International Digital Accountability Council reported that many apps are sending unencrypted transmissions which is completely contrary to best practices [80]. Therefore, it is essential to encrypt all communications from the mobile device to the destination.
• **Encryption:** Although the proximity information usually consists of pseudo-anonymous IDs, the registration information can be susceptible. Users typically have to provide name, address and phone number during the registration. Such information requires encryption before storing in the server. Furthermore, some of the protocols use absolute location (e.g., GPS data) to track users’ location and proximity. Such information is highly privacy sensitive and should be treated accordingly.
• **False-positive:** The reporting of the COVID-19 positive follows two main approaches: i) voluntary, where individuals update their status as COVID positive, and ii) authority-triggered, meaning government officials (often health professionals) update the status of the individuals. The first approach is particularly vulnerable to exploitation. If a group of ill-motivated people update their status as COVID positive, there could be public chaos, making the app ineffective.
• **Privacy:** The contract tracing process cannot be completely anonymous. In our review, we have found that large number of protocols have used pseudo-identifier to protect the privacy of the individuals. The personally identifiable information (PII) is stored in a database which can only be accessed by health professionals. However, in some distributed architecture-based protocols, end users have the capability to check their contract proximity data without registration. It provides partial privacy. In these practices, individuals with COVID positive results need to upload their contract tracing information (e.g., Bluetooth beacon) whereas others will only verify their proximity against that data.

### C. Recommendations

**FIGURE 17.**
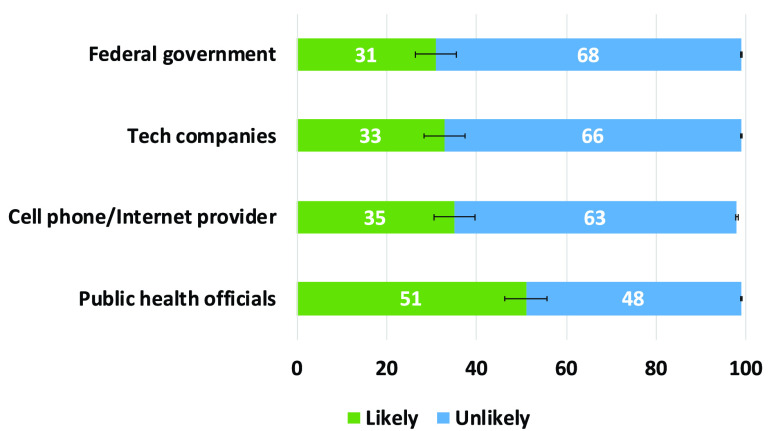
Percentage of US adults who are likely/unlikely to use COVID-19 app.

With all the efforts put by the researchers, practitioners, and governments around the world, the general sentiment among the ordinary citizens (in USA) is still negative as illustrated in Figure 17 [81]. The success of any contact tracing app will be heavily dependent on the trust of the citizens. Therefore, efforts must be in place to increase the trust level, and thus the adoption rate of the society. In the following, we put down a few recommendations that can help to build users’ confidence and the level of trust, which in turn would make contact tracing more universal and effective.

• **Challenge of Wide-Scale Adoption:** The main obstacle with the current app-based contact tracing mechanism is smartphone penetration level, specifically in developing counties. Counties like India and Bangladesh, where smartphone penetration is quite low (25.3% and 18.5% respectively [82]), smartphone app-based approach will not be very effective. High coverage of contact tracing apps is vital for effectiveness. There have been few mobile phone network based initiatives; however, they are not very beneficial in terms of identification of the contacts. Low-cost wristband based proximity detection could be used in low-socioeconomic areas with the cooperation of community health workers.
  In addition, the vulnerable seniors who are not digitally connected but are at higher risk from COVID-19 needs special consideration. For example, Singapore government has introduced TraceTogether Tokens, which sends Bluetooth signals to other tokens, or smartphones with the app, and each one uses a personalised QR code [70].
• **Limitation of Bluetooth Technology:** It has been observed that due to the privacy concern and granularity issues, Bluetooth has been the most used technology for the contact tracing app [83]. However, it has issues with proximity measurement. It cannot differentiate if there is any physical object between two phones. For example, if two people live in two apartments separated by a brick wall, the app will still detect it as being nearby. This phenomenon is disquieting for highly congested apartments in cities. Researchers have recommended computing the intersection of the user’s trajectories to have more robust contact tracing apps [84]. However, the effectiveness of this approach is still doubtful. Moreover, since it is designed based on RSSI only and the expected range of Bluetooth is significantly higher than the distance recommended for social distancing, this approach potentially collects lot more contacts than required, making apps to generally produce a higher false positive. More refined calibrations around RSSI and the inclusion of transmitted signal strength in Apple-Google API may overcome this problem, but further studies investigating this area would be necessary [85].
• **Build Trust between Government and Citizen:** Building trust between citizen and state is vital for such apps to be successful. These apps can be easily turned into a surveillance tool unless the citizen’s privacy is legally protected. Mission creeping is another major concern to civil societies. Although some countries such as India and South Korea have made contact tracing apps mandatory for their citizens, the majority of the countries with contact tracing apps have opted for voluntary participation. Different countries, including Australia, have passed laws to control the exposure to the data to various parties, specifically to the law enforcement authorities. However, this pandemic is evolving and legal experts should continuously explore better protection for the ordinary people to help build trust between the state and its citizens.
• **Transparency:** Many users expressed concerns regarding the nature of the permission apps are using [86]. Governments should make the whole initiative more transparent in terms of the legal framework and the app. One of the approaches government or other app provider can take is to open source the entire code, including the server side, of the respective apps. Additionally, app providers can invite independent security auditors or penetration testers to independently review the security and privacy measures of the app in protecting the privacy and security of the users.
• **Implementation Details:** It has been observed that contact tracking apps often do not work properly due to implementation glitches [87]. For example, the COVIDSafe app has been widely criticised for not delivering the results as expected [87]. The problems are mainly in software implementation, e.g., difficulties in detecting nearby devices by locked iPhones, an interoperability issue when sharing data between iPhone and Android phones.
• **Distributed Ledger Technology:** Many protocols and app studied in this paper are centralised, and the health authority/government controls the access. Centralised approaches also suffer more from denial of service attacks. Distributed ledger technology such as blockchain may help to mitigate such attacks [88]. It also helps to build trust in the system due to its transparency and immutability properties [89].
• **Data Inter-operability Among Different Apps:** In near future, when the restriction will be lifted and the people can travel from one country to another country, different apps will need to be inter-operable so that different apps can talk to each other and health authority can retrieve the data.
• **Balance Between Privacy and Data Usability:** Keeping a good balance between the privacy of the individuals and the usability of the data is very vital to successfully track COVID patients and their contracts. It is always a trade off. If the system allows to register without PII, it would be too difficult (if not impossible) for the health professionals to track new contact. On the other hand, if PII of all citizens is stored in a single database, their is a high risk of compromise or misuse.

At any given time, contact tracing may become a serious act against individual’s privacy. During a pandemic if it is necessary to use a contact tracing app to save lives and reduce fatality, a ‘good app’ must have a privacy-preserving architecture beneath as we have seen in some discussions earlier in the protocol and app reviews. Despite Bluetooth being less accurate, it seems to be more privacy-friendly than any other existing technologies. Its performance can be improved using Kalman filters and artificial intelligence techniques to more accurately identify the potential presence of humans in the vicinity. A good app must grant the users the ability not to disclose their condition after testing positive for any reason. The decision to use the app and disclosing one’s information should always come from the users, the app must not allow third parties to dictate over users’ decisions. It is also important that a good app works decentrally and stores information locally. Users should be allowed to delete their data and stop using the app at any time. If they decide to disclose their condition by submitting their data to any central server, the architecture of the app must ensure that users cannot be traced back, the data transferred between users’ phone and the server over a secured medium and the submitted data is removed without keeping any backup after a certain period preferably within two to four weeks of submission.

## VI. Conclusion

The COVID-19 pandemic is a public health crisis that reminds us of the importance of being prepared for such an emergency. Ever since the novel coronavirus began to spread in China, researchers around the globe proposed contact tracing methods in the form of protocols and smartphone apps. Contact tracing, however, is not something invented recently instead has been practised for years in various ways to combat pandemics. The most recent approach, the COVID-19 contact tracing, utilises smartphone-based and wireless network-assisted applications. In this paper, we critically analysed the underlying technologies, protocols and those apps proposed for this pandemic. The objective of the assessment was to identify their shortcomings against a set of threats and other matrices derived from our investigation and possible ideal functionalities that protocols could potentially offer, together presented as a taxonomy at the beginning of the article. We then provided three comprehensive reviews for the underlying technology, protocols and the contact tracing apps. These reviews formed the basis for the critical studies, presenting two summary tables demonstrating the corresponding protocols and the threats that they mitigate. This review finally explicated the existing gaps in the proposed protocols and how they can be improved to combat future pandemics.
